# Denoising framework for X-ray absorption spectroscopy data

**DOI:** 10.1107/S1600577526001712

**Published:** 2026-03-24

**Authors:** Tomas Aidukas, Ilnura Usmanova, Benjamín Béjar Haro, Maarten Nachtegaal, Adam H. Clark

**Affiliations:** a Center for Photon Science Paul Scherrer Institute Villigen Switzerland; b Swiss Data Science Center Paul Scherrer Institute Villigen Switzerland; c Department of Chemistry and Applied Biosciences ETH Zürich Zürich Switzerland; University of Malaga, Spain

**Keywords:** XAS, denoising, X-ray absorption spectroscopy, Gaussian process, autoencoder

## Abstract

X-ray absorption spectroscopy (XAS) is a powerful tool for probing the structural and electronic properties of materials, but its analysis is often challenging due to the low signal-to-noise ratio of the XAS spectra. Denoising of XAS spectra is particularly challenging because the measured spectral features span a broad range of feature widths. Such spectral feature variability is called non-stationarity. XAS spectra also suffer from energy-dependent noise and are often acquired using non-uniform energy sampling. While a broad range of denoising methods exists, they underperform when dealing with non-stationary and non-uniformly sampled signals. We introduce a novel stationarity warping approach, which transforms XAS spectra into a domain where they appear stationary, resulting in greatly improved denoising performance. This warping approach can be combined with any denoising method. We also implemented advanced denoisers based on Gaussian process regression and a convolutional autoencoder. All of the denoising and stationarity warping methods are packaged into a Python-based denoising software called *XASDenoise*, which provides a modular, easy-to-use denoising functionality of XAS measurements. Our benchmarking shows that stationarity warping consistently enhances spectral feature preservation and noise suppression across a diverse range of XAS datasets and is applicable to any denoising method.

## Abstract 2

We introduce a high-performance Python-based XAS denoising package called *XASDenoise*, which implements our newly developed stationarity warping method and advanced denoising techniques based on Gaussian process regression and a convolutional autoencoder.

## 1. Introduction

X-ray absorption spectroscopy (XAS) is a measurement technique that can provide insights into the structural, electronic, and chemical properties of materials (Yano & Yachandra, 2009 ▸; Chantler *et al.*, 2024 ▸; Newville, 2014 ▸; Van Bokhoven & Lamberti, 2016 ▸). From a signal perspective an XAS spectrum contains energy-dependent features such as sharp and broad peaks. Whether specific material properties can be determined from such data will depend on how well the spectral features can be resolved and differentiated from noise. In practice, noise can strongly affect data pre-processing steps such as normalization and background subtraction, both of which are prerequisites for downstream analysis. In extended X-ray absorption fine structure (EXAFS) analysis, noise directly impacts the reliability of fitted structural parameters (Booth & Hu, 2009 ▸). In X-ray absorption near-edge structure (XANES) linear-combination fitting, noise reduces the accuracy of component fitting and can lead to incorrect estimation of components (Rojsatien *et al.*, 2023 ▸). More generally, spectral decomposition methods, such as multivariate curve resolution analysis, are sensitive to noise, which greatly affects component identification and separation (Martini & Borfecchia, 2020 ▸). As a result, denoising has the potential to improve the robustness and quality of various XAS analysis methods.

A common source of noise is the limited photon flux of the X-ray source, resulting in a poor signal-to-noise ratio (SNR) (Abe *et al.*, 2018 ▸). Noise is particularly problematic in fluorescence XAS measurements of low-concentration samples (Yano & Yachandra, 2009 ▸; Chantler *et al.*, 2024 ▸) and quick-XAS measurements, where a spectrum across a broad X-ray energy range can be acquired in under a second (Müller *et al.*, 2016 ▸). While quick-XAS is essential for time-resolved studies of fast catalytic reactions, it often requires reduced X-ray flux to limit beam-induced damage, resulting in increased noise levels. While noise is present in measurements using high-flux synchrotron X-ray sources, it becomes the limiting factor for XAS measurements using low-flux laboratory-based X-ray sources. Therefore, denoising has the potential to improve the performance of laboratory-based XAS measurements.

**Figure 1:**
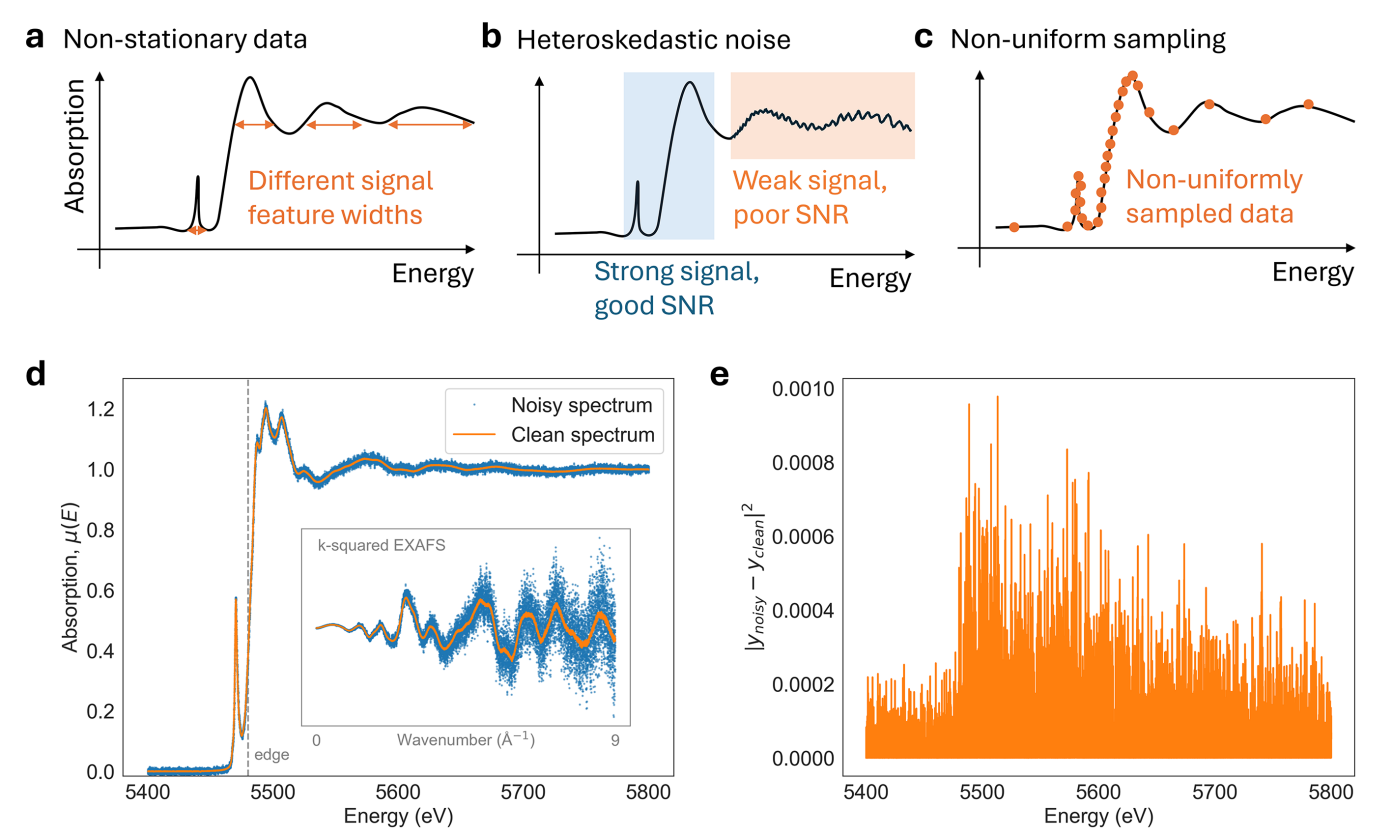
An XAS data set is difficult to denoise effectively since it is (*a*, *d*) non-stationary (varying signal feature widths); it also contains (*b*, *e*) heteroskedastic noise (noise whose properties vary across the spectra), and (*c*) it can be non-uniformly sampled.

One property of XAS data which makes denoising challenging is its *non-stationary signal structure* [Figs. 1 ▸(*a*) and 1(*d*)], meaning that the characteristic features of the absorption spectrum change across the energy range. In this work, non-stationarity refers to variations in the structure widths/sharpness of the signal itself, rather than to changes in the noise statistics. This non-stationarity is most evident when comparing the XANES region, which contains sharp and narrow features near the absorption edge, with the EXAFS region, which consists of broader, weaker, and more oscillatory features at higher energies. As a result, denoising parameters that are optimal for noise suppression in the EXAFS region can oversmooth fine features in the XANES region if applied uniformly across the spectrum.

In addition to non-stationary signal structure, XAS spectra are further complicated by the presence of *heteroskedastic noise* [Figs. 1 ▸(*b*) and 1(*e*)] and by non-uniform sampling. Unlike imaging modalities that acquire uniformly sampled data by a pixelated detector, XAS spectra are measured over a user-defined energy range that can be sampled sparsely, non-uniformly, or adaptively [Fig. 1 ▸(*c*)].

Over the past few decades, a wide range of methods have been developed for denoising of one-dimensional signals, such as XAS spectra. Classical approaches such as the Savitzky–Golay filter (Schafer, 2011 ▸), Whittaker smoothing (Eilers, 2003 ▸), Butterworth low-pass filters (Selesnick & Burrus, 1998 ▸), wavelet transforms (Luo *et al.*, 2012 ▸), and empirical mode decomposition (Rilling *et al.*, 2003 ▸) leverage assumptions about signal smoothness or sparsity. These methods are popular due to their simplicity, low computational cost, and ease of use. However, they often lack adaptivity and can struggle with signals that exhibit non-stationary or heteroskedastic noise characteristics, all of which are commonly observed in XAS data.

More recent advances include denoising approaches based on dictionary learning (Yue *et al.*, 2023 ▸), deep neural networks (Klein *et al.*, 2022 ▸), and probabilistic frameworks such as deep Bayesian models (Kang *et al.*, 2021 ▸), which aim to model data uncertainty and structure through hierarchical and flexible parameterizations. While these methods show improved performance, particularly for complex noise structures, they typically require large training datasets, careful tuning, and often generalize poorly across varying experimental conditions. Hence, more efficient and easier-to-use denoising methods are desired for XAS signal denoising.

An alternative but closely related approach is denoising via Gaussian processes (GPs) (Williams & Rasmussen, 2006 ▸), which also operate within a Bayesian framework. GPs offer strong uncertainty quantification and are particularly well suited for denoising irregularly sampled and highly noisy data, which is commonly encountered in XAS. However, standard GP models assume stationarity, which is problematic when dealing with non-stationary and heteroskedastic XAS signals (Snelson *et al.*, 2003 ▸). To address this, several extensions have been proposed, including domain splitting (Gramacy & Lee, 2009 ▸), deep Gaussian processes (Damianou & Lawrence, 2013 ▸), and learned input warpings (Snelson *et al.*, 2003 ▸).

While effective, the current denoising methods are often computationally expensive, complex to implement, sensitive to hyperparameter choices, and often not generalizable (Liu *et al.*, 2020 ▸; Hensman *et al.*, 2013 ▸). As a result, XAS analysis is typically performed using classical denoising approaches such as Butterworth filtering (Manceau *et al.*, 2014 ▸), Fourier filtering (Ross *et al.*, 2024 ▸), or principal component analysis (Vogt *et al.*, 2018 ▸). Additionally, publicly available spectroscopic denoising software packages such as *SpectroChemPy* (Travert & Fernandez, 2025 ▸) offer traditional approaches, such as Whittaker (Eilers, 2003 ▸), Savitzky-Golay (Schafer, 2011 ▸), and moving averaging filters. The disconnect between existing modern signal denoising algorithms and their implementation for XAS data analysis highlights the need for an easy-to-use, high-performance denoising framework.

In this work, we introduce a fully automated and easy-to-use XAS data denoising software package called *XASDenoise* (Aidukas, 2025 ▸), which consists of several innovations. One key innovation is our data-driven input warping approach called *stationarity warping*, which transforms the non-stationary XAS signals into a domain where they appear stationary. Such a transformation can greatly improve the denoising performance of XAS spectra, and is applicable to any denoising method.

Another innovation is the development of a GP-based XAS data denoiser, which is packaged into an easy-to-use method with many of the important steps performed automatically. Combined with the *stationarity warping* approach, our GP denoiser performs exceptionally well across a broad range of XAS spectra, and it is especially effective when dealing with highly noisy XAS signals.

We also introduce a supervised denoising method based on a convolutional autoencoder architecture, which outperforms all unsupervised denoising methods present within our denoising pipeline. A common limitation of convolutional autoencoder-based methods is their poor generalization to data acquired under different experimental conditions, such as using different energy scanning steps. By using the *stationarity warping* method, the XAS signals can be transformed onto a grid where the autoencoder denoiser is most effective, enabling our implemented autoencoder denoiser to generalize across diverse XAS sampling conditions. The optimal architecture and training procedure are included in our *XAS­Denoise* software package.

In summary, our framework provides a robust and flexible denoising methodology for XAS spectra.

### 1.1. Paper structure

Our paper is structured as follows. First, in Section 2, we introduce the rationale and methodology of the stationarity warping approach and also provide an overview of our *XASDenoise* denoising pipeline. In the ensuing sections, we describe each step of our pipeline in detail. In Section 3 we describe the necessary signal pre- and post-processing methods. In Section 4 we define, motivate, and describe our stationarity warping methodology, including estimation of the signal warping function. Next, in Section 5, we describe the need for signal interpolation onto a uniformly sampled grid and the associated challenges. Later, in Section 6, we introduce the denoising methods implemented within our denoising pipeline. They include traditional unsupervised denoising methods such as Butterworth, wavelet, *etc*. (Section 6.1) and our proposed approach to automatically select the optimal denoising parameters. Our framework also supports GP regression for denoising (Section 6.2) and a convolutional autoencoder (Section 6.3) trained on our experimental XAS dataset. Finally, in Section 8 we provide a quantitative and qualitative evaluation of our denoising pipeline and compare the implemented denoising methods. We also demonstrate that the application of stationarity warping significantly improves the denoising performance of any denoising method. These findings are concluded in Section 10. Technical implementation details of our *XASDenoise* software package are provided in Appendix *A*[App appa].

## 2. Non-stationary signal denoising pipeline

**Figure 2:**
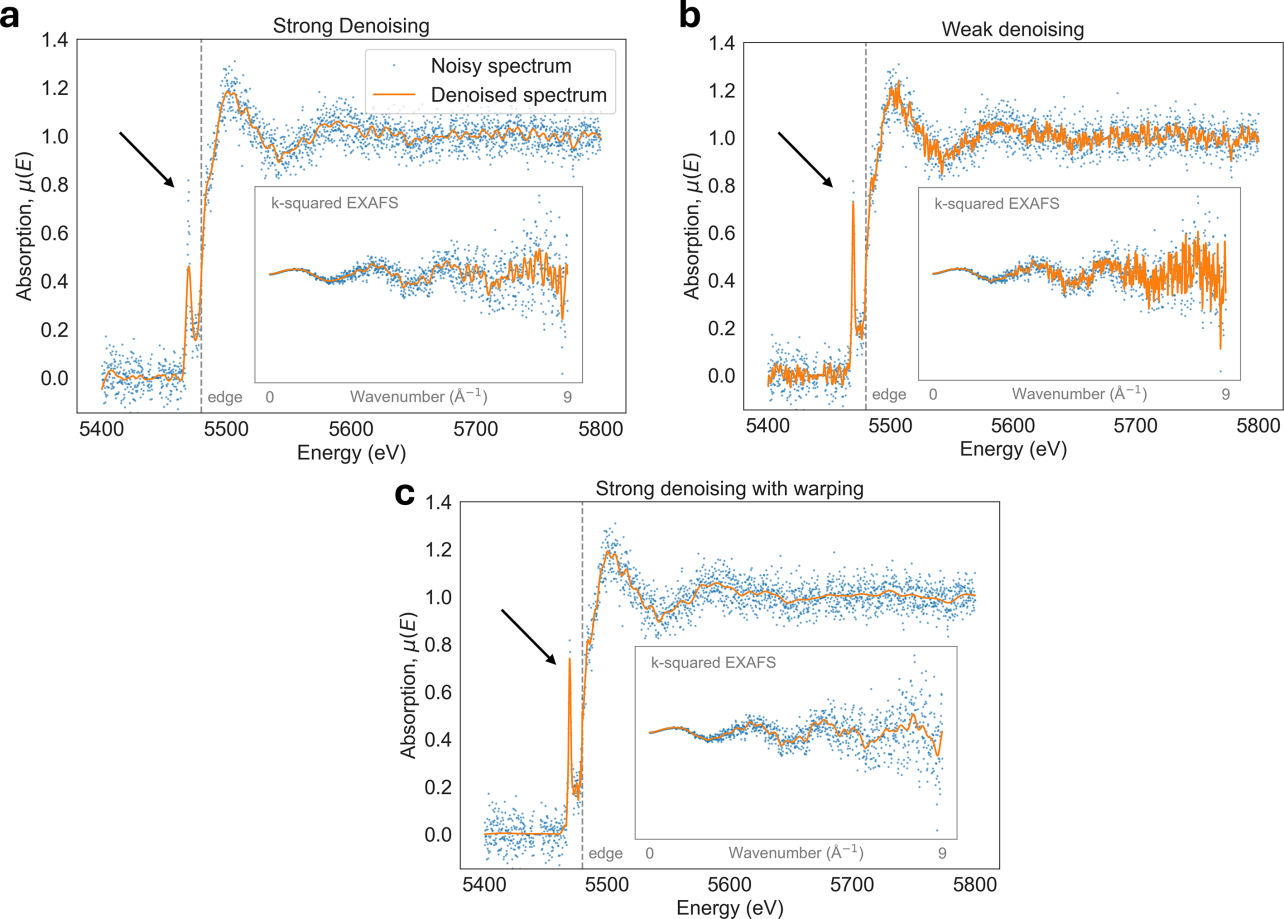
(*a*) With strong signal filtering (*e.g.* using a Butterworth filter), the EXAFS region is denoised well at the expense of smoothing out the sharp pre-edge peak. (*b*) By reducing the denoising strength, the pre-edge peak sharpness is maintained while the EXAFS region remains noisy. (*c*) By using interpolation-based data warping, not only is the noise in the EXAFS region highly suppressed but the sharp pre-edge features in the XANES region are also maintained.

Denoising of non-stationary signals, such as XAS data, is challenging because classical denoising methods struggle to preserve spectral features of varying width and sharpness. This issue persists across all denoising methods based on signal low-pass filtering, which removes high-frequency noise arising from photon counting statistics, electronic interference, and mechanical instabilities (Abe *et al.*, 2018 ▸). Therefore, XAS data denoising often comes at the expense of sharp spectral feature oversmoothing, as illustrated in Fig. 2 ▸(*a*). While oversmoothing can be avoided by reducing the denoising filtering strength, this comes at the expense of poor denoising performance, as shown in Fig. 2 ▸(*b*). With our newly introduced non-stationarity warping method, we can achieve optimal denoising performance and preservation of sharp spectral features, as shown in Fig. 2 ▸(*c*). Non-stationarity is also a problem for the GP-based denoisers, since GP regression uses stationary kernels (*e.g.* Matérn) (Williams & Rasmussen, 2006 ▸) which fail to model the diverse range of features in the XAS signal.

### 2.1. Denoising of non-stationary signals

**Figure 3:**
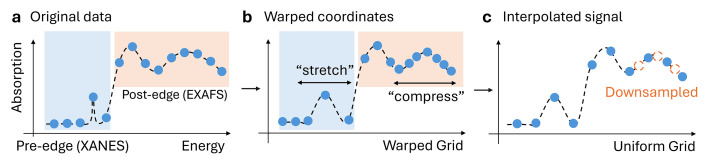
The goal of warping is to transform the data in (*a*) to a domain in (*b*) where it appears stationary using coordinate warping. For denoising methods that do not take datapoint coordinates into account, the signal must be interpolated onto a uniformly sampled grid in (*c*).

To mitigate the signal processing issues associated with signal non-stationarity, we propose to use a signal transformation method called *warping*. In the context of XAS signals, the warping transformation can be regarded as a mapping of any point from the original energy domain *x* into a new domain *x*_warped_. The goal of warping is to stretch and compress the XAS signal features *y* = *f*(*x*), such that the signal is transformed from (*x*, *y*) into a domain (*x*_warped_, *y*) where it becomes more stationary, see Figs. 3 ▸(*a*) and 3(*b*). We formally define warping in Section 4 and propose several methods to estimate the stationarity mapping functions. Warping has been successfully used for GP regression (Snelson *et al.*, 2003 ▸), which allows GPs to model non-stationary signals. Therefore, warping plays a key role in our GP-based XAS denoising method (and all of the other denoising methods presented in this manuscript), since the signals (*x*, *y*) can be denoised in the warped domain (*x*_warped_, *y*).

However, most denoising methods do not take into account the input coordinates *x* and instead assume that the signals *y* are uniformly sampled. Therefore, the use of warping for most denoising methods requires an additional signal interpolation step that transforms the non-stationary signal *y* onto a warped and uniformly sampled grid *x*_uniform_. Through interpolation, we will obtain a warped signal *y*_warped_ as shown in Fig. 3 ▸(*c*).

After the above data transformation steps, we consider the transformed signal to be stationary and can apply one of the denoising methods within our pipeline. These methods include traditional denoisers, a GP-based denoiser, and a convolutional autoencoder-based denoiser trained on experimental data described in Section 7.2. Once denoising is complete, the warping is undone by mapping the signal back to the original input domain. Fig. 2 ▸ demonstrates that, by using stationarity warping, denoising methods are able to minimize noise and also preserve spectral features of the XAS signal.

### 2.2. Overview of the denoising pipeline

**Figure 4:**
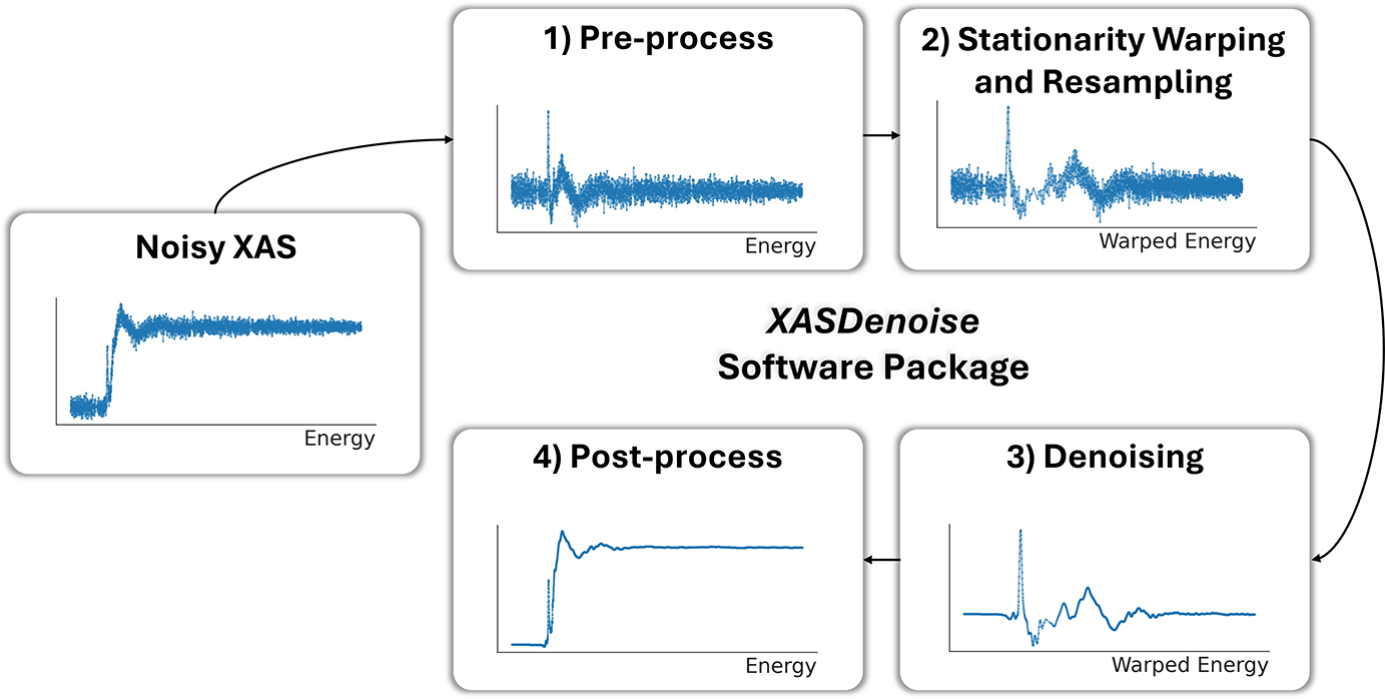
Schematic illustration of our *XASDenoise* denoising pipeline.

The denoising pipeline illustrated in Fig. 4 ▸ is designed to improve the SNR of XAS data by integrating preprocessing, data warping, and automated parameter tuning prior to denoising. We summarize the workflow in the following five modular steps:

(i) Data pre-processing: remove the absorption edge-step from the spectrum to obtain a zero-mean signal, estimate heteroskedastic noise, and identify the absorption edge location.

(ii) Stationarity warping: estimate a coordinate transformation function that maps the original energy grid to a new domain (*e.g.* wavenumber) where the XAS signal becomes stationary.

(iii) Resampling on a uniform grid: many denoisers require data points to be sampled on a uniform grid, which can be achieved by interpolating the signal onto a warped and uniformly sampled grid. This step is done only for denoisers that assume uniformly sampled data.

(iv) Denoising: apply a selected denoising method to the warped signal.

(v) Post-processing: revert the signal to its original coordinate domain and add back the edge-step to produce the final denoised spectrum.

Each step is modular, making the framework flexible and compatible with various denoising algorithms. We demonstrate that the above procedure improves the performance of all unsupervised and supervised denoising methods. In the following sections, we describe each step in detail.

## 3. Data pre-/post-processing

The purpose of this initial data processing step is to preprocess the XAS data and compute all of the necessary arrays for subsequent steps within the denoising pipeline. An important preprocessing step is the estimation and removal of the XAS edge-step function to obtain a zero-mean signal. In the context of XAS data analysis, our background subtraction is analogous to the extraction of the EXAFS signal χ(*E*) from the measured absorption spectrum μ(*E*). The edge-step is estimated by fitting a spline function with several knots, similarly to the approach described by Newville (2013 ▸). Edge-step subtraction ensures that the resulting signal has zero mean, which can improve the denoising performance of some methods, such as the GP denoiser introduced later. It also allows the warping and denoising steps to operate on the oscillatory structure of the signal without interference from the low-frequency background.

In addition to edge-step removal, heteroskedastic (energy-dependent) noise is estimated using a sliding window approach. Within each window, the noise is assumed to follow a Gaussian white noise distribution with fixed variance. This noise is calculated by computing the deviations of the data from a polynomial fit, which is an estimate of the underlying clean signal. We chose a linear fit model due to its robustness against overfitting compared with other higher-order polynomials. Ultimately, an initial estimate of the energy-dependent noise is obtained for each of the sliding windows. This energy-dependent noise is later used as a prior in the Gaussian process denoiser and is further refined during model training.

Another important parameter computed during preprocessing is the absorption edge energy *E*_0_, which is required for the physics-based data warping methods. The edge energy is estimated as the point where the estimated edge-step function reaches half of its maximum intensity (provided that the XAS signal is normalized). In the denoising pipeline, this value is used to apply region-specific warping methods for pre-edge and post-edge regions.

Once denoising is complete, all preprocessing steps are reversed to reconstruct the signal on the original coordinate grid and ensure that the denoised signal remains compatible with any downstream analysis that expects data in the original coordinate system. This includes converting the signal from the warped domain back to the original coordinate space *x*. If the signal was interpolated onto a uniform grid *x*_uniform_, then the denoised signal must first be interpolated back onto the original grid *x*. In most cases, the original grid is denser than the uniform grid used during processing, which can lead to minor interpolation artifacts. However, if the denoised signal is sufficiently clean, these artifacts are typically negligible and do not compromise the quality of the final output. Lastly, we add back the absorption edge-step function that was subtracted prior to XAS signal denoising.

## 4. Stationarity warping

The key feature and novelty of our denoising pipeline is the *stationarity warping* method, which significantly improves the performance of any denoising method. While warping can be used for a broad range of applications, such as finding a transformation to match two signals (Bloemberg *et al.*, 2013 ▸), we define *stationarity warping* as a transformation of the input domain to a domain where the signals appear more stationary. For example, sharp features (*e.g.* edges) can be stretched, and smooth regions compressed, yielding a stationary signal with uniform feature widths [see Figs. 3 ▸(*a*) and 3(*b*)].

### 4.1. Definition of stationarity warping

Our goal is to transform the input coordinates *x* of a signal *y* = *f*(*x*) into a new set of coordinates *x*_warped_, such that the signal appears stationary. Here, both *x* and *y* contain a discrete set of *N* measurements 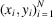. Using XAS spectra as an example, *x*_*i*_ would correspond to the X-ray energy *E*_*i*_, and *y*_*i*_ would correspond to the noisy absorption spectrum *y*_*i*_ = μ(*E*_*i*_) + ε, where μ(*E*_*i*_) is the absorption coefficient and ε is additive random noise. For a given signal, we call *x* the *input* and *y* the *output*.

We define *input warping* as a non-linear, strictly monotonous transformation function 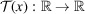, that maps each point *x*_*i*_ from the original input domain *x* to a warped domain 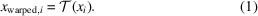
Strict monotonicity ensures that the ordering of input points is preserved, and only the spacing between points changes. The transformation is reversible, and the outputs *y* themselves remain unchanged. After warping, the XAS spectrum becomes 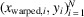.

The warping transformation changes the relative distances between input points, which is key to making the signal appear stationary. The next section will describe how to construct such a warping function.

### 4.2. Compression factor and warping function construction

The effect of any warping transformation 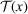 can be described using a *compression factor* τ(*x*), which quantifies how much the spacing between neighboring points is scaled under the transformation.

To obtain the relationship between 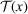 and τ(*x*), let *x*_1_ and *x*_2_ be two adjacent input values. After applying 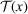, the distance between them changes from |*x*_1_ − *x*_2_| to
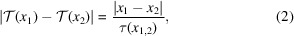
where 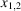 = 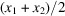 is the midpoint.

This defines τ(*x*) as a local scaling factor for the input space, which we can approximate as the inverse of the gradient norm,
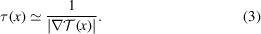
To ensure that the transformation is valid and invertible, we require 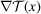 > 0 for all *x*, which guarantees strict monotonicity and prevents singularities. For example, a function like 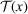 ≃ 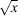 must be regularized near *x* → 0 where its derivative diverges.

This relationship between 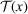 and τ(*x*) allows for two complementary strategies for constructing the transformation function 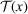:

(i) Known transformation function: if the transformation 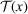 is known from prior knowledge (*e.g.* from physical modeling), we can compute *x*_warped_ directly from 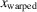 = 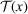. Moreover, we can also compute the compression factor τ(*x*) from the gradient of 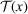 using equation (3), which gives an interpretable measure of how local distances are scaled by the transformation, up to a shifting constant.

(ii) Data-driven compression function: if 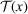 is not known *a priori*, we may define a compression factor τ(*x*) directly from the signal (*e.g.* based on estimated smoothness or curvature). In this case, the warping function is computed by integrating the inverse of τ(*x*),
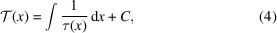
where *C* is a constant offset. Once 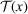 is computed, it can be used to transform the input via 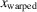 = 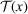.

This unified formulation highlights that *x*_warped_ is always computed from a transformation function 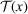, whether that function is known or derived. The compression factor τ(*x*) acts as a bridge between local signal structure and the transformation, making it a central tool for both analysis and construction of warping functions.

### 4.3. Stationarity warping functions

To construct a suitable stationarity warping function 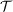, we introduce three methods:

(i) A physics-based approach where 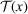 is obtained directly from EXAFS theory.

(ii) A data-driven approach where 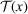 is estimated from signal smoothness.

(iii) A hybrid approach combining both methods.

Each method produces a different 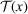 and thus a different warped input domain. After warping, the new input–output pairs (*x*_warped_, *y*) can be passed into any denoiser that uses both *x* and *y* as input.

**Figure 5:**
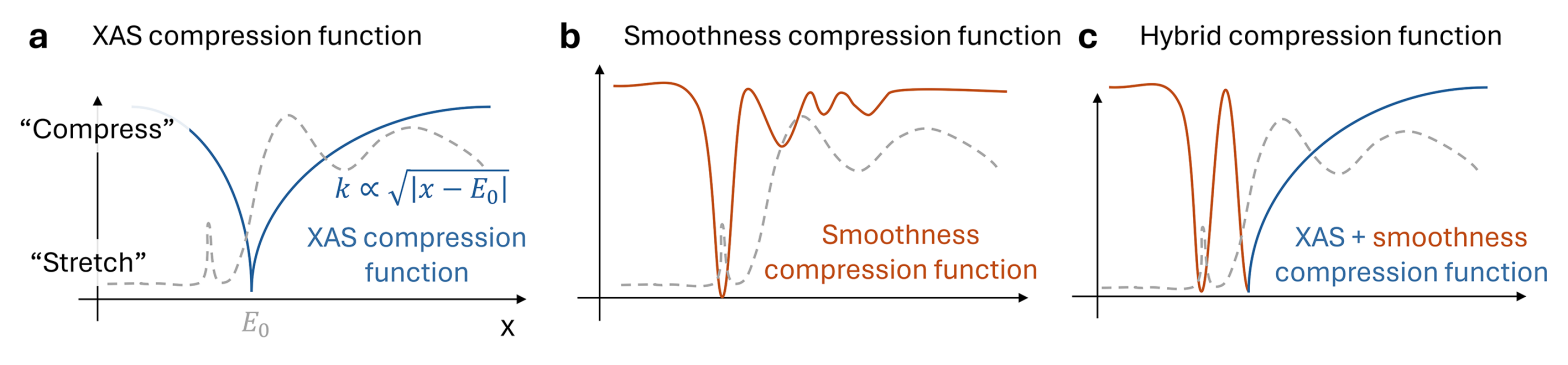
Illustration of compression factors τ(*x*) for each transformation function 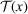 obtained via three different warping strategies: (*a*) physics-based relationship between energy and wavenumber, (*b*) signal-smoothness-based warping, (*c*) hybrid combination of both. The compression factors illustrate how the input energy grid *x* is stretched and compressed for a given XAS spectrum.

To visualize what effect 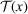 has for a given grid *x*, we can look at the corresponding compression factors τ(*x*) for each transformation function. Figs. 5 ▸(*a*)–5(*c*) illustrate how the input domain is stretched and compressed by a given transformation function.

### 4.4. XAS warping function

In the EXAFS region of XAS, the signal can be modeled as a superposition of sinusoidal oscillations with different frequencies, as described by the EXAFS equation. These oscillations are naturally defined in the wavenumber domain *k*, where the signal appears approximately stationary. However, XAS data are typically acquired in the energy domain *E*, which is non-linearly related to *k*-space (wavenumber) according to the following expression,
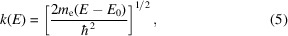
where *m*_e_ is the electron mass, ℏ is the Planck constant, *E* is the XAS energy and *E*_0_ is the absorption edge energy.

This square-root relationship compresses the higher-energy oscillations, such that features that are stationary in *k*-space appear non-stationary in energy space. To compensate, we define a transformation function 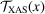 that maps the energy grid *x* = *E* to a symmetrized k-space domain,
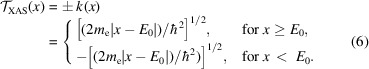
This transformation can be used directly to compute 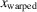 = 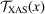. The resulting domain stretches the sharp XANES features near the edge while compressing slowly varying regions further from the edge, making the entire signal appear more stationary. The resulting compression factors are illustrated in Fig. 5 ▸(*a*).

**Figure 6:**
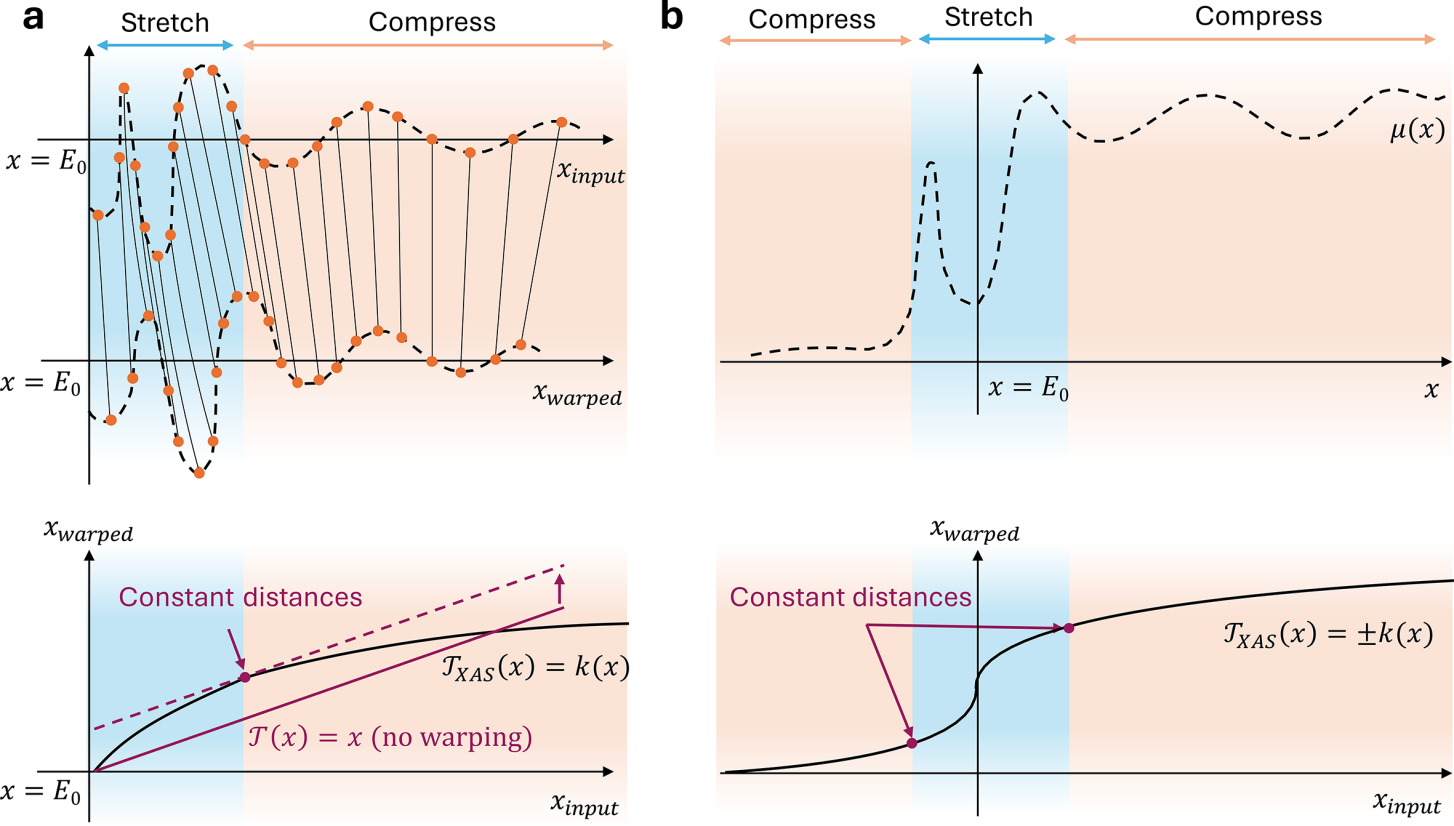
(*a*) Illustration of input warping and how the regions get stretched/compressed. Without warping the function 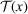 = *x* does not change the location and distances between the points, since the compression factor is equal to 1. If we use 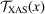 = *k*(*x*), the resulting compression factor would compress regions further away from the absorption edge *x* = *E*_0_. (*b*) Illustration of the extended XAS warping function to the pre-edge and post-edge regions of an XAS spectrum.

The compression/stretching behavior can be interpreted via the associated compression factor described in equation (3). In combination with the XAS warping function described in equation (6), the local scaling between the neighboring energy points *x* after the transformation will be described as 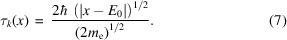
This equation shows that the signal compression factor will be higher for higher energies. The resulting stretching and compression of the signal for *k*-space scaling is shown in Fig. 6 ▸. However, because the compression factor is defined only up to a shifting constant, we need a reference to interpret whether a region is effectively stretched or compressed. A transformation function that returns the unwarped signal can be used as a reference, *i.e.*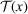 = 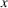. The energy *x* at which the compression factors for warped and non-warped transformation functions are equal will define the inflection point between signal stretching and compression as illustrated in Fig. 6 ▸.

The introduced *XAS warping function*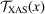 is well suited for XAS signals, because in the *k*-space domain all of the spectral features appear similarly broad. This is desirable for denoising because it allows the denoisers to treat all spectral features consistently.

### 4.5. Smoothness-based warping function

To generalize stationarity warping beyond the EXAFS region, we define a transformation function 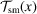 estimated directly from the signal itself.

We define local signal smoothness using the estimated curvature of the underlying clean signal *f*(*x*). Specifically, we apply a sliding-window second-order polynomial fit to approximate the Hessian norm ∇^2^*f*(*x*). Larger curvature indicates sharper features, while smaller curvature corresponds to flatter regions. The window must be small enough to accurately estimate the local curvature, yet large enough to mitigate the effects of noise.

We define the compression factor as the inverse square root of the estimated Hessian norm,
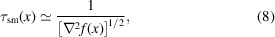
so that sharper features are sampled more densely (less compression), while smoother regions are compressed. An example of the compression factors is shown in Fig. 5 ▸(*b*).

Finally, we construct the warping transformation via integration, 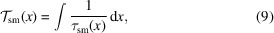
and compute 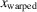 = 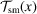. This method yields a transformation function that adapts directly to the structure of the observed signal.

### 4.6. Hybrid warping function

While the smoothness-based warping function is effective in the XANES region, signal smoothness estimation can be insensitive to weak and noisy features in the EXAFS region. In contrast, the *k*-space warping function is physically grounded and performs well for EXAFS but lacks interpretability in the XANES region.

To combine the strengths of both methods, we introduce a hybrid strategy:

(i) Use 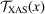 in the EXAFS region (post-edge).

(ii) Use 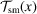 in the XANES region (pre-edge).

(iii) Blend them in the transition region near the absorption edge *E*_0_.

The resulting compression factors are shown in Fig. 5 ▸(*c*), combining the advantages of both warping functions.

## 5. Resampling onto a uniform grid

Most traditional denoising methods—such as Butterworth, Gaussian, or moving average filters—do not make use of the coordinate values *x*, assuming that the signal values *y* = *f*(*x*) are sampled on a uniform regular grid. For these methods, coordinate warping alone is insufficient, since after the warping the data points remain on a highly non-uniform grid.

One way to deal with the above issue is to interpolate the signal *y* itself from the warped grid *x*_warped_ onto a uniformly sampled grid *x*_uniform_ as shown in Fig. 3 ▸(*c*). This transformation makes the signal appear stationary to the denoiser and leads to significantly improved denoising performance, as illustrated in Fig. 2 ▸. However, interpolation of noisy data is non-trivial because it can introduce artifacts such as correlated noise (Rybicki & Press, 1992 ▸). To minimize interpolation artifacts, we need both a suitable interpolation method and a well aligned uniform grid *x*_uniform_. To avoid information loss when interpolating the noisy signal onto a sparser grid *x*_uniform_, we use a custom two-step interpolation.

First, we create a uniform grid *x*_uniform_. A safe and robust approach is to create *x*_uniform_ that spans the same range as *x*_warped_ but has a grid spacing *h* set to the *maximum* spacing found in *x*_warped_. Although this undersamples the densest regions, it prevents oversampling in sparse, high-noise regions, which could introduce correlated noise. To avoid using anomalously large step sizes (*e.g.* due to missing data), we instead select the grid spacing for *x*_uniform_ as the 90th percentile of the absolute finite differences of the warped sampling locations, *h* = percentile(|*x*_warped,*i*+1_ − *x*_warped,*i*_|, 90). Therefore, most of the analyses presented here use a uniform grid that contains fewer points than the warped grid, effectively downsampling the signal prior to denoising.

Secondly, we split the signal (*x*_warped_, *y*) into discrete bins of width 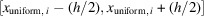 centered around every uniform grid point *x*_uniform,*i*_. Then, the measurement points (*x*_warped_, *y*) within each bin are averaged, producing a single unique averaged point per bin 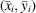. However, the averaged location 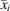 might not exactly match the center position of each bin *x*_uniform,*i*_. To correct for the possible mismatch, the averaged signal 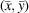 is interpolated onto the uniformly sampled grid *x*_uniform_, resulting in a uniformly spaced and binned signal 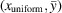.

Our proposed interpolation approach resamples the signal on a uniformly sampled grid while also providing two distinct advantages compared with regular interpolation. First, it mitigates information loss in densely sampled regions by applying binning before interpolation. Second, it generates a uniform grid *x*_uniform_ that is sparser than the original grid *x*_warped_, which prevents oversampling and minimizes the introduction of correlated noise.

## 6. Denoising methods

This section describes the denoising methods currently implemented within our pipeline, which include traditional denoisers, a GP-based denoiser, and a convolutional autoencoder denoiser.

### 6.1. Traditional denoising methods

By traditional denoisers, we refer to well established and widely used filtering methods that take a noisy signal *y* as input and return a denoised signal 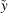. The current implementation includes the following methods (see Appendix *B*[App appb] for more details of each method): *moving average filter, Gaussian filter, Savitzky-Golay filter, median filter, Butterworth filter (low-pass), wavelet denoiser, total variation denoiser*.

Most traditional denoisers operate solely on the signal intensity values *y*, without using the corresponding coordinate values *x*. Instead, these methods assume that the input signal is uniformly sampled, an assumption that holds in many cases, for example, when data are acquired using detectors with evenly spaced pixels (*e.g.* image sensors). However, this assumption becomes limiting when working with warped, non-stationary signals. In such cases, the signal must first be interpolated onto a uniformly sampled grid in the warped domain to satisfy the requirements of these denoisers (see Section 5).

We also introduce an automated parameter optimization strategy for traditional denoisers in the Appendix *B*1 [App appb], based on minimization of a total-variation (TV) loss. This approach enables consistent and efficient parameter selection across all denoisers, providing a fair comparison of denoising methods and ensuring robust performance on diverse XAS datasets.

### 6.2. Gaussian process regression

**Figure 7:**
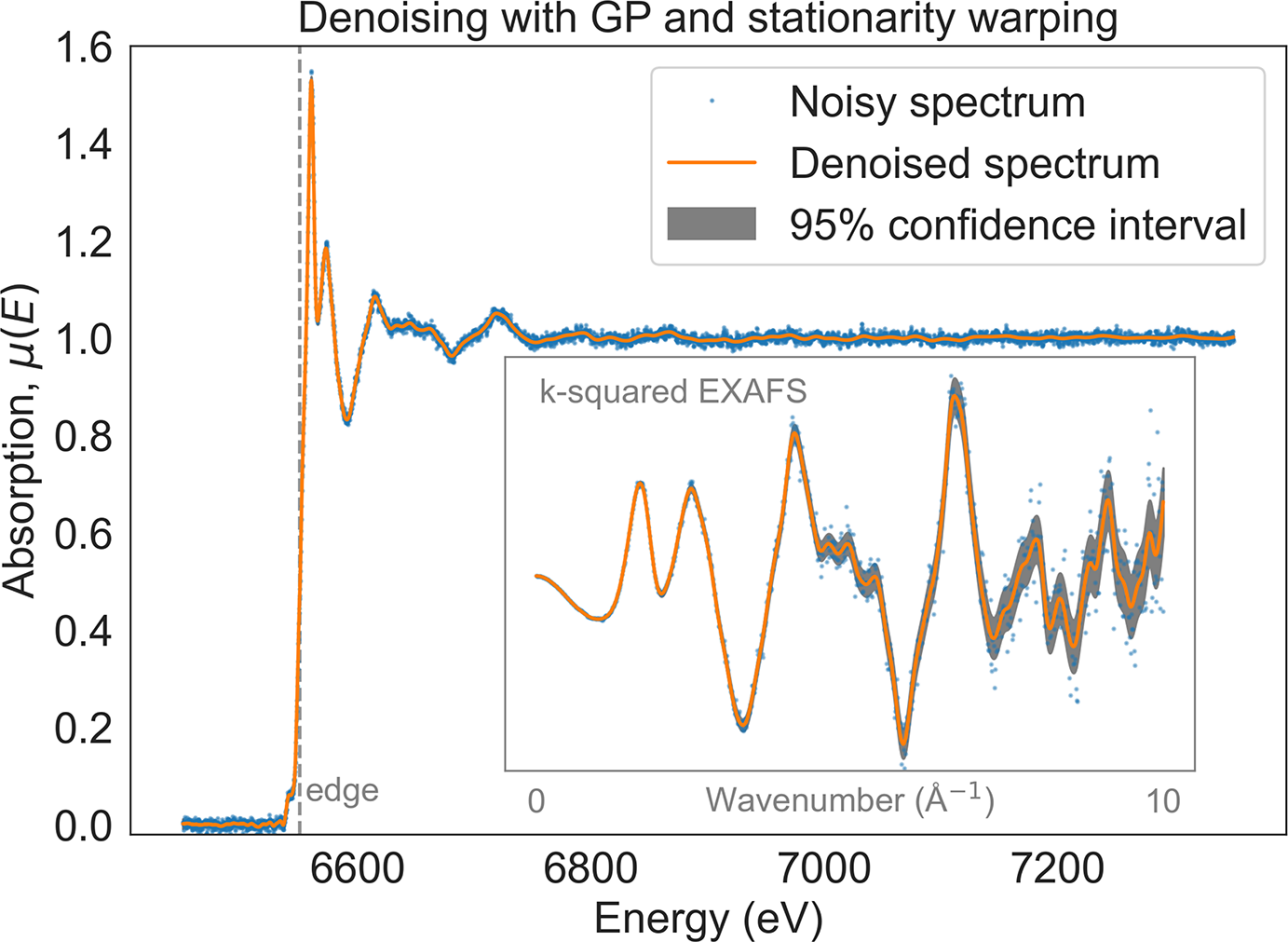
Example of GP regression (with a Matérn-5/2 kernel) performed on an Mn *K*-edge LiMn_2_O_4_ XAS spectrum using our denoising pipeline introduced in Section 2. In addition to signal denoising, GPs can also estimate the denoising uncertainty as shown by the 95% confidence interval.

GP regression is a flexible, non-parametric denoising method. Unlike parametric methods, the model complexity of GPs grows with increasing number of measurements. This allows GPs to improve their denoising capabilities as more measurements become available. In contrast, parametric denoising methods assume that a clean underlying spectrum can be expressed using a model whose capacity remains the same regardless of the amount of data. Moreover, GPs introduce only general assumptions about the spectrum through a kernel function, which encodes correlations between neighboring data points. Through this kernel, the GP conditions its predictions not only on adjacent measurements but also on more distant points, depending on the characteristic correlation length scale. For example, if the kernel encodes smoothness over a 5 eV energy window, the GP will average out noise over that range while preserving essential spectral features such as absorption edges and fine-structure oscillations. Importantly, GPs also provide an uncertainty estimate for each predicted point, as illustrated in Fig. 7 ▸. Further implementation details are provided in Appendix *C*[App appc].

Given noisy observations 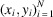, we model the measurements as 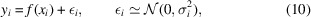
where the term ε_*i*_ represents measurement noise at each data point *x*_*i*_, modeled as a Gaussian random variable 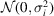 with zero mean and variance 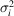. We can model the underlying function *f*(*x*) as a Gaussian process 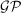, defined by a mean function μ_GP_(*x*) and covariance (kernel) function *k*_GP_(*x*, *x*′),
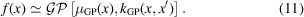
By subtracting the edge-step function from the XAS signal, we can assume that the mean is close to zero μ_GP_(*x*) = 0.

The GP model has several important hyperparameters, including the choice of kernel and its parameters (*e.g.* the lengthscale), the noise prior σ^2^(*x*), and the mean function. In our case, the stationarity warping procedure allows us to model the signal using a stationary GP with a single global kernel length scale. For denoising, we use a Matérn kernel with smoothness ν = 5/2. Modern libraries like *GPyTorch* (Gardner *et al.*, 2021 ▸) automatically estimate the lengthscale by maximizing the marginal likelihood (a statistical criterion that measures how well the model explains the observed data while accounting for model complexity). The most critical modeling choice, then, becomes how to set the prior for the heteroskedastic noise σ^2^(*x*), which we describe in Appendix *C*5[App appc].

### 6.3. Supervised convolutional autoencoder

Supervised learning approaches have shown strong promise for spectroscopic data denoising (Pate *et al.*, 2021 ▸), leveraging the fact that spectra often exhibit repeatable patterns across compounds, elements, and oxidation states. In this section, we describe our convolutional autoencoder-based denoiser and architectural design choices. We also describe how we trained it using a *Noise2Noise* (Lehtinen *et al.*, 2018 ▸) strategy using only noisy data pairs without the need of clean reference data.

#### 6.3.1. Training data curation and processing

We trained the convolutional autoencoder using noisy–noisy data pairs, following the *Noise2Noise* approach (Lehtinen *et al.*, 2018 ▸), which does not require clean ground truth data. This method assumes each pair consists of independent realizations of the same signal corrupted by uncorrelated noise. Compared with noisy–clean training data pairs, the *Noise2Noise* approach is better suited to our data, where we measured a collection of noisy spectrum repeats in quick-EXAFS mode (Müller *et al.*, 2016 ▸). While averaging of noisy repeats could provide an approximate clean reference spectrum for constructing noisy–clean pairs, the resulting ‘clean’ signal is often an imperfect estimate. Instead, with the *Noise2Noise* approach, we avoid the need for perfectly clean reference data and are less affected by temporal experimental drifts during repeated measurement of noisy spectra.

As mentioned in Section 7.1, all of our XAS spectra undergo identical preprocessing steps during both training and inference. First, we normalize every spectrum such that the pre-edge region absorption is around zero and the post-edge region is centered around one (Calvin, 2013 ▸). Secondly, we subtract the XAS edge-step function to ensure all spectra are approximately zero-mean, as described in Section 3. This edge-step subtraction was done not only for training but also during data denoising. In doing so, all of the spectra used for training consist of oscillations centered around zero, and no data normalization during the training step is required.

Our training dataset contains nearly 60000 unique training samples, as described in Section 7.2. All of our spectra were acquired under a wide range of experimental conditions, resulting in substantial variation in energy sampling grids and noise levels. To ensure consistency across samples, all spectra were downsampled onto a common uniform energy grid as described in Section 7.1. We chose downsampling instead of interpolation to avoid introducing correlated noise. However, downsampling creates only an approximately uniform grid, since there will always be small discrepancies in grid sampling between the desired and originally measured grids. While such discrepancies are unavoidable between experimental measurements, we believe that they can improve the autoencoder generalization capabilities by exposing the model to a broader range of spectral feature positions and widths.

Similarly, while our stationarity-warping procedure (Section 4) enhances denoising of non-stationary signals, we found that an autoencoder trained directly on the original, non-warped data generalizes better when denoising both warped and non-warped spectra. We hypothesize that without warping the autoencoder is able to learn a broad range of spectral features and generalize to unseen spectra. Warping, on the other hand, aims at making all spectral features similarly broad, reducing the generalization capabilities of the convolutional autoencoders.

#### 6.3.2. Model architecture and training

**Figure 8:**
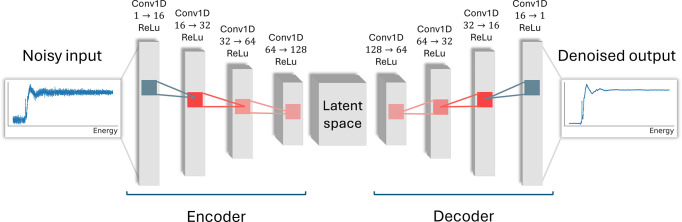
Convolutional autoencoder architecture consisting of a symmetric encoder and decoder, which was trained for XAS data denoising. During encoding, the input spectrum is mapped to a latent representation that captures the dominant spectral features shared across the dataset, while uncorrelated noise is largely suppressed. The decoder then reconstructs a denoised spectrum from this latent representation.

Our model is a 1D convolutional autoencoder consisting of an encoder and a symmetric decoder (Fig. 8 ▸). The use of a convolutional autoencoder for XAS denoising is motivated by the fact that spectral variations in XAS can be described by a relatively sparse set of recurring features. The autoencoder exploits this property by learning a compact latent representation that captures the dominant spectral structure shared across many spectra, while suppressing noise that does not follow these spectral patterns. Related ideas of using latent representations and dimensionality reduction have been applied to XAS data analysis (Tetef *et al.*, 2021 ▸).

The encoder maps the input spectrum into a high-dimensional latent space using *L* = 4 convolutional layers with channel dimensions [16, 32, 64, 128], kernel size *k* = 9, and reflect padding to reduce edge artifacts. The decoder mirrors this structure using transposed convolutions, with output padding removed. After each convolutional layer we apply a rectified linear unit (ReLU) activation to introduce non­linearity, allowing the network to capture complex, nonlinear relationships in the spectral features while maintaining stable gradients during optimization.

We trained the model using a weighted mean squared error (MSE) loss. To emphasize the weak EXAFS oscillations beyond the absorption edge, we scale the loss using 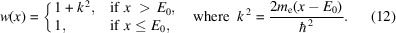
In conventional EXAFS analysis, *k*^2^ weighting is commonly applied to enhance high-*k* oscillations while damping the low-*k* region where the atomic-like background is not well defined. Instead, our 1 + *k*^2^ scaling function is used to preserve the low-*k* signal while amplifying high-*k* oscillations, allowing weak EXAFS features at large *k* to influence the autoencoder training. In doing so, the weak EXAFS signal after the absorption edge *x* > *E*_0_ becomes equally important as the strong XANES region around the absorption edge *E*_0_.

During evaluation, we used a metric of RMSE scaled by the estimated noise level, RMSE/σ, to enable a uniform comparison of denoising performance across spectra with different noise levels, as described in Section 8.1. In principle, we could also use a scaled loss function during training, such as MSE/σ^2^. While this would unify the training and evaluation loss metrics, it would introduce several drawbacks. Firstly, scaling the loss by the noise would down-weight the contribution of high-noise spectra, resulting in poorer denoising performance of the highly noisy data. Secondly, division by small noise values σ^2^ can destabilize training convergence due to the amplified gradient variance. Instead, we trained the autoencoder using the standard MSE loss, which yields more stable optimization and better generalization across the wide range of noise conditions present in our experimental XAS dataset.

The network was trained for 2000 epochs using the Adam optimizer (Kingma & Ba, 2017 ▸) with a learning rate of 10^−3^ and batch size 128. To handle zero-padded spectra, the spectra within each batch were cropped to the shortest sequence within each batch. By masking the data, we achieve two things. Firstly, artificial zero values are not seen by the network. Secondly, it acts as a data augmentation step, since each randomly assembled batch is cropped to the smallest spectrum energy range at every training epoch, leading to better generalization.

## 7. XAS dataset for training and testing

The experimental XAS data used for training and benchmarking were acquired at the SuperXAS operando spectroscopy beamline in Swiss Light Source, Paul Scherrer Institute (Bugaev *et al.*, 2024 ▸). At SuperXAS, the XAS data are acquired in quick-EXAFS mode, where the monochromator is rapidly rotated to quickly sweep the desired energy range. The high-speed energy scanning enables time-series measurements under *operando* and *in situ* conditions. Moreover, we can also acquire many noisy repetitions of *ex situ* samples, which do not undergo any temporal changes. Such an acquisition provides us with multiple noisy spectrum repeats, which can be averaged to produce a noise-free spectrum estimate.

Our XAS dataset contains approximately 300 XAS reference spectra, covering a broad range of elements and chemical compounds. The resulting spectra span a broad range of noise levels that one can expect to encounter in a typical XAS experiment, making this dataset ideal for denoising method benchmarking and supervised denoiser training.

In the following sections we will explain how our dataset was pre-processed for autoencoder training and denoising method testing.

### 7.1. XAS dataset processing

Given the diverse range of spectra within our dataset, we performed the following pre-processing steps to unify all spectra for training and benchmarking purposes. First, all spectra were normalized such that the pre-edge baseline was set to zero and the post-edge absorption was scaled to one (Calvin, 2013 ▸). Secondly, we cropped all spectra to 100 eV before the absorption edge and 800 eV after the edge. The absorption edge location was selected as the halfway point of the rising absorption edge, such that XAS spectra of all chemical compounds have equally long pre-edge and post-edge energy regions. Thirdly, to ensure consistent energy sampling, all spectra were downsampled onto a uniformly sampled energy grid with an energy resolution of 0.18 eV. Such energy resolution corresponds to 5000 points per spectrum within the 900 eV energy range used for cropping. Spectra with shorter pre-edge and post-edge regions were zero-padded such that each array contains 5000 points and a mask was created to identify the padded values. Padding enables computationally efficient data storage in a single multi-dimensional array, which is also required for efficient supervised denoiser training.

### 7.2. Training and testing

The resulting XAS dataset contains multiple spectra *y*_*c*, *i*, *t*_, consisting of *c* = [1,…, *C*] XAS spectra associated with a unique chemical compound. Each compound contains *N* discrete energy measurements *i* = [1,…, *N*] at each time instance *t* = [1,…, *T*]. While our spectra were measured as a time-series using the quick-EXAFS acquisition mode, they can be equivalently interpreted as repeated measurements of the same underlying spectrum acquired under identical conditions. In this context, time averaging corresponds to averaging over multiple repeats, which leads to improved SNR. Therefore, throughout the manuscript, we will refer to a single spectrum time-instance as a noisy spectrum ‘repeat’. The average of these noisy spectrum repeats will be treated as an estimate of the ‘clean’ or ‘true’ spectrum.

In total, we have *C* = 261, which was split into training and testing/benchmarking sub-datasets consisting of *C*_test_ and *C*_train_ compounds. First, compounds *c* were grouped into ten noise levels using a percentile approach. Then, XAS spectra within a given noise level were split into training and testing sub-datasets at random using a 10/90 data split. This was done to ensure that relatively rare noisy spectra would feature with equal probability within the training and testing datasets. After splitting, the testing/benchmarking dataset contained *C*_test_ = 37 XAS compounds, while the training dataset contained *C*_train_ = 224 XAS compounds for supervised denoiser training.

For each compound *c* we can extract multiple clean–noisy data pairs from the collection of repeated noisy measurements. The clean signal is estimated by averaging all of the repeated measurements 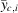 = 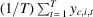 and assigning it to every noisy repeat *y*_*c*, *i*, *t*_. The result is a data pair 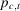 = 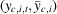.

For supervised denoiser training, we extracted 56307 noisy–clean data pairs 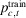 from *C*_train_ = 224 compounds, each containing hundreds of repeated noisy measurements.

For testing/benchmarking, we extracted a single data pair from each compound, resulting in 37 clean–noisy pairs. For the noisy signal, a single noisy repeat *t*_1_ was selected, such that at *t*_1_ the difference between the noisy and clean spectra is minimal, 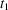 = 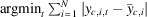. This was done to minimize the mismatch between the clean and noisy signals due to experimental drifts during acquisition of repeated noisy measurements. The result is a data pair 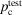 = 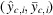, where 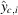 = 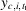 is the noisy measurement.

## 8. Denoising pipeline analysis

In this section, we will introduce our benchmarking framework, evaluate the denoising performance improvements delivered by our warping methodology, and compare the performance of various denoising methods. Within this section, the term ‘warping’ refers either to energy grid warping (for GP denoisers) or to signal interpolation onto a uniform and warped grid (for traditional and autoencoder-based denoisers). Additionally, we demonstrate the superior performance of Gaussian process and autoencoder denoisers compared with traditional methods.

We evaluated warping and denoising performance on experimentally acquired XAS data, where both high- and low-SNR spectra were obtained experimentally using a fast-scanning (quick-EXAFS) acquisition mode (see Section 7). For each XAS spectrum compound, multiple rapid scans were collected under identical experimental conditions, yielding an ensemble of statistically independent noisy realizations of the same spectrum. A high-SNR reference (‘true’) spectrum was constructed by averaging many repeated measurements, while individual repeats serve as low-SNR (‘noisy’) spectra. This approach is statistically equivalent to varying integration time in conventional step-scanning experiments and enables direct evaluation of denoising accuracy and stability.

**Figure 9:**
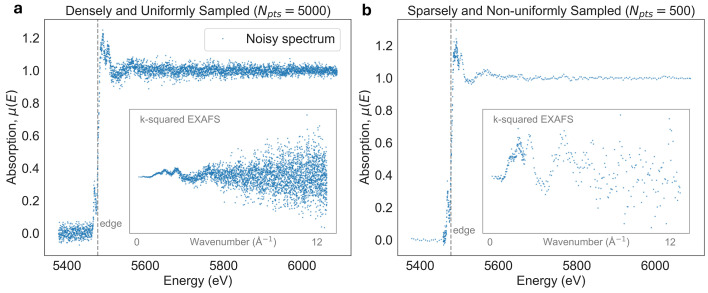
Experimental XAS data acquisition often produces two types of XAS spectra such as the (*a*) dense and uniformly sampled spectra during quick-EXAFS data acquisition and (*b*) sparse and non-uniformly sampled step-scan spectra.

While both step-scanning and quick-EXAFS scanning can produce the same spectra, in practice experimental limitations impose constraints on how the spectra are sampled. Therefore, we evaluated warping and denoising performance on two types of experimental XAS datasets shown in Fig. 9 ▸. The first type of data is *densely and uniformly sampled*, which spans an energy range of 900 eV in steps of 0.18 eV, resulting in around 5000 data points per spectrum (see Section 7.1). This dataset was acquired experimentally in quick-EXAFS mode (Bugaev *et al.*, 2024 ▸), providing uniformly and densely sampled XAS signals, which is unique to only a few XAS beamlines around the world.

The second type of data is *sparsely and non-uniformly sampled* (see Section 8.4), where the energy range is scanned by rotating the monochromator in discrete steps rather than a high-speed continuous sweep as in quick-EXAFS. Since the stop-and-go motion of the step-scanning approach results in significant motion overhead, XAS data are typically acquired on sparse and non-uniformly sampled energy grids compared with quick-EXAFS. To compensate for the signal sparsity in some areas, the exposure time is increased, leading to large SNR variations across the whole energy range. This means that the denoising methods must not only cope with non-uniform sampling but also with highly heteroskedastic noise.

### 8.1. Benchmarking framework

As described in Section 7.2, our testing dataset contains *c* = [1,…, 37] clean–noisy data pairs 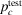 = 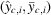. To benchmark the denoising methods, we denoised the noisy measurement 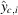, resulting in the denoised signal 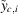 = 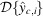, where 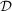 is a denoising operator. Here, the measurements 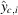 are assumed to be the XAS spectra μ(*E*), acquired in energy-space. As mentioned in Section 3, we also perform absorption edge subtraction as a denoising pre-propcessing step, which is similar to producing the so-called χ(*E*) signal for EXAFS analysis.

The denoising performance was quantified by computing the root mean squared error (RMSE) between the denoised and clean, high-SNR spectra,
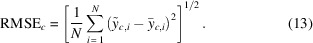
To compare denoising performance across compounds *c* with varying noise levels σ_*c*_, we instead use the normalized RMSE (nRMSE),
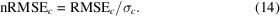
Here σ_*c*_ is computed as the standard deviation of the residual between noisy and clean spectra 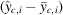. Once normalized, the nRMSE values can be interpreted as follows:

(i) nRMSE = 1: denoised signal is similar to the noisy signal (no improvement).

(ii) nRMSE < 1: denoised signal is approaching the estimated clean signal (values of zero imply perfect denoising).

(iii) nRMSE > 1 denoised signal is of worse quality than the noisy input (oversmoothing).

To obtain a single performance score for each denoiser 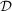, we average the normalized errors across all denoised chemical compounds *c*. In doing so, we obtain a *mean normalized error* for each denoiser: 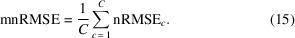
This mean normalized error was used to quantify relative denoising method performance within the manuscript.

### 8.2. Comparison of warping methods

Stationarity warping requires the use of a specific coordinate warping function, which we described in Section 4.3. In this section, we evaluated the performance of each warping and denoiser method combination using uniformly sampled XAS datasets, described in Section 7.1 and illustrated in Fig. 9 ▸(*a*).

**Figure 10:**
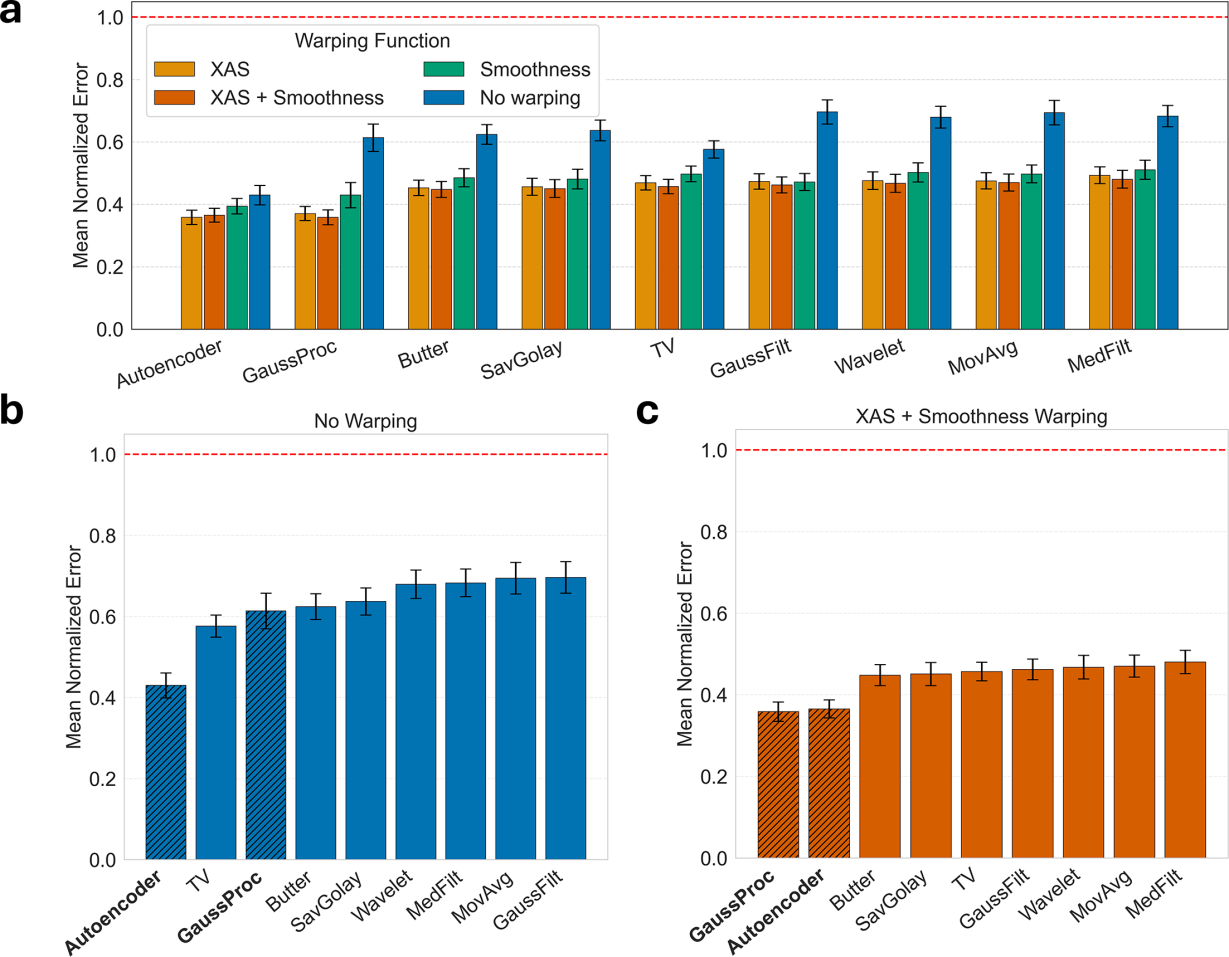
(*a*) Comparison of warping methods across a broad range of denoisers shows that the overall denoising performance is greatly improved when data warping is performed. (*b*) Without warping, the denoising performance is suboptimal, especially for the GP denoiser, which performs similarly to other traditional denoising methods. (*c*) With stationarity warping, the denoising performance drastically improves for all methods, and classical denoisers can perform as well as modern denoisers without warping. The red dashed line indicates how similar the denoised signal is to our ‘clean’ reference spectrum, with values below unity representing improved quality.

Results shown in Fig. 10 ▸(*a*) demonstrate that the performance of all denoisers improves similarly across the tested coordinate warping functions. The slight performance variations indicate that the selection of a coordinate warping function will depend on the signal and denoising method. For example, spectra with strong pre-edge features can benefit from a smoothness-based warping function, because the physics-based XAS warping function has a physical meaning only for the post-edge EXAFS region and is ill-defined for the pre-edge. Therefore, the selection of a good warping function is a critical step in improving the denoising performance.

The denoiser performance comparison between non-warped and optimally warped signals is shown in Figs. 10 ▸(*b*) and 10(*c*). Based on Fig. 10 ▸(*b*), the denoising performance variations are greater when warping is not used, indicating that the denoisers struggle to achieve optimal performance across a broad range of chemical compounds. Moreover, the GP denoiser performs relatively poorly compared with others, because GPs assume that the underlying signals are stationary, resulting in poor GP optimization convergence.

Once warping is used, the performance of all methods improves significantly, as indicated by Fig. 10 ▸(*c*), with the autoencoder and GP denoisers outperforming the traditional methods. Warping makes data appear more stationary, which is required for good GP convergence. Moreover, once the signals are interpolated onto a uniformly sampled warped grid, traditional denoisers are able to denoise a broad range of spectral features with a single set of denoising parameters. Without warping, the methods would denoise some features while oversmoothing others, as we demonstrated earlier in Fig. 2 ▸. More importantly, warping makes the denoising performance equally good across all traditional denoising methods, making even simple averaging filters perform exceptionally well.

Moreover, looking at Figs. 10 ▸(*b*)–10(*c*), the autoencoder denoiser performance improved when it was applied to warped data. This is surprising because the autoencoder denoiser was trained on non-warped data, yet it performs better when warping is used. As we discussed in Section 6.3, it is likely that training on non-warped data exposes the autoencoder to a broad range of spectral features of various widths. This way, the encoder learns how to denoise a broader range of features, whereas with warping the training would reduce the scope of feature widths. These claims are based on our observation that an autoencoder denoiser trained on warped data performs more poorly than one trained on non-warped, originally acquired datasets. In general, the autoencoder denoiser generalized well for data sampled differently compared with the one used for training.

### 8.3. Comparison of denoising methods

**Figure 11:**
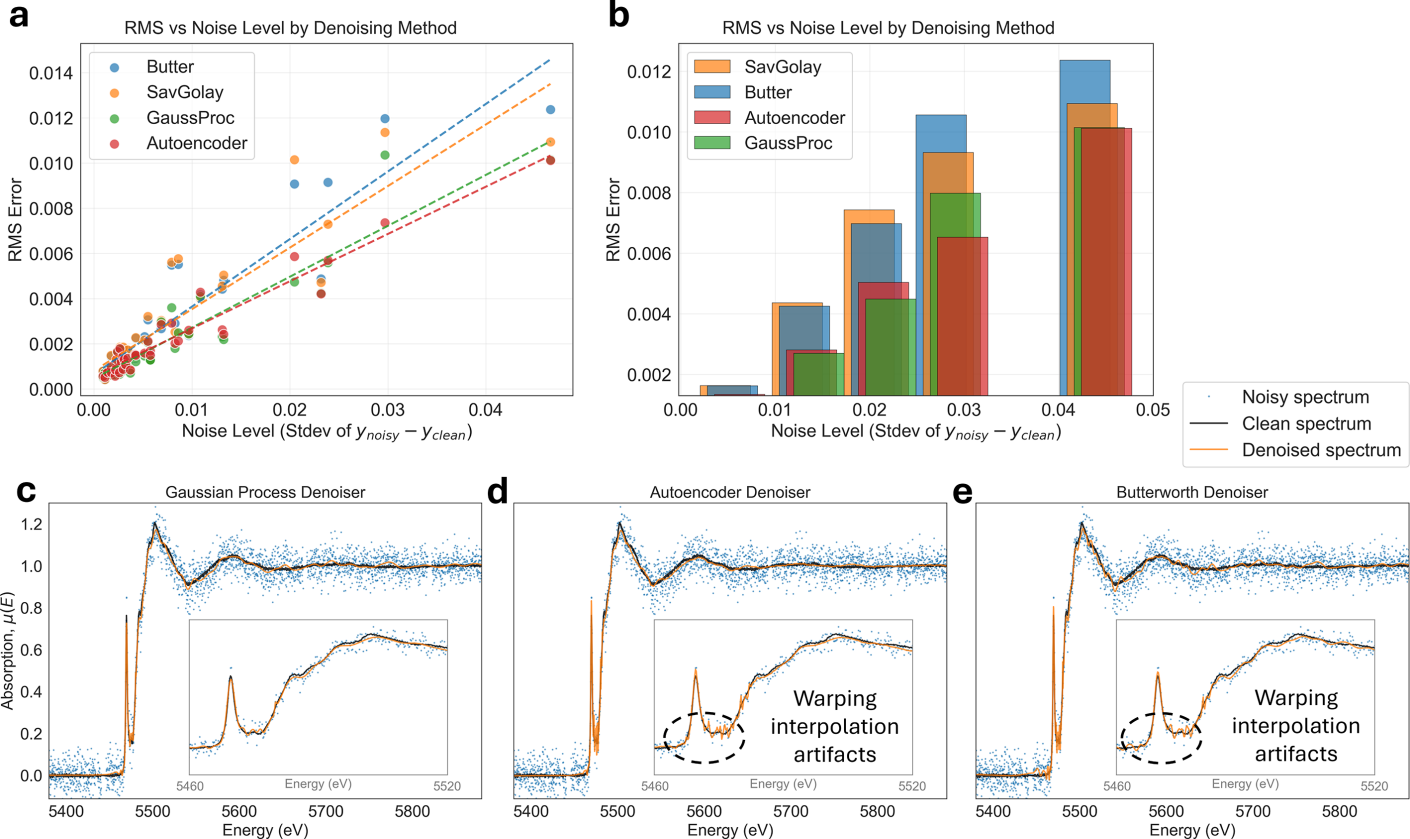
(*a*) The XAS spectra within our benchmarking dataset have a range of noise levels, with only a few of them being very noisy. (*b*) By grouping the compounds into noise bins, we can better understand how denoising methods perform across various signal-to-noise levels. At the lowest noise levels, all methods perform similarly but, once the noise level increases, the discrepancies also increase. Overall, the Gaussian processes and autoencoder denoising methods on average perform better across most noise level bins and especially when dealing with the noisiest signals. The noisiest compound within the dataset is the V *K*-edge BiVO_4_ XAS spectrum shown in (*c*)–(*e*). We also highlight regions that were ‘stretched out’ during warping, resulting in overfitting of the noise, which is avoided by the GP denoiser.

To better understand the denoiser performance across various XAS compounds, we investigated how the top four denoising methods from Fig. 10 ▸(*c*) perform across various noise levels. Figs. 11 ▸(*a*) and 11(*b*) reveal that the XAS signals used for benchmarking are relatively clean, with only a small minority having larger noise levels. Unfortunately, very noisy spectra are rare, with only one to two spectra per noise bin, limiting the statistical significance of our conclusions. Overall, the denoising performance of GP and autoencoder denoisers is best for every signal noise level. If we look at the denoising performance of the noisiest compound in Figs. 11 ▸(*c*)–11(*e*), qualitatively, the differences are relatively small. However, minor interpolation artifacts appear near the absorption edge, where the signal was ‘stretched’ by the warping process, leading the denoiser to interpret small deviations between neighboring noisy XAS data points as genuine spectral features rather than noise. This issue is suppressed for the autoencoder denoiser and non-existent for GPs, since it does not require signal interpolation for stationarity warping. Therefore, both the autoencoder and GP denoisers are less affected by interpolation artifacts introduced through the warping process.

### 8.4. Denoising of sparse, non-uniformly sampled data

So far, we have shown that data warping can modify the sampling of XAS spectra and significantly improve denoising performance. This implies that the sampling pattern used during data acquisition itself can strongly influence the quality of denoising. Therefore, in this section, we will investigate how the acquisition sampling affects denoising performance by using *sparsely and non-uniformly sampled* data, as illustrated in Fig. 9 ▸(*b*).

We generated this dataset by taking our original uniformly sampled dataset described in Section 7.1 and resampling it onto the following non-uniformly sampled grid:

(i) Pre-edge region: up to 20 eV before the absorption edge *E*_0_, sampled using 5 eV energy steps.

(ii) Edge region: ±20 eV around the absorption edge *E*_0_, dense sampling of 0.18 eV (same as the dense data used for benchmarking).

(iii) Post-edge region: from 20 eV after the absorption edge *E*_0_, sampled linearly in *k*-space using 0.05 Å^−1^ steps.

The resulting sparse and non-uniform dataset representing step-scan data acquisition contains spectra around 500 points long, which is 10× sparser than the quick-EXAFS dataset. To mimic longer exposure times within the sparser regions, for each energy point we averaged *N*_*t*_ = *r*/0.18 eV repeated noisy measurements where *r* is the energy sampling around the selected point. This approach maintains the original SNR around the edge region since the energy sampling is the same in both uniformly and non-uniformly sampled spectra, while the SNR is greatly increased for the sparse pre-edge region.

To perform data warping for both traditional and autoencoder-based denoisers, we first interpolate the XAS spectra onto a uniformly sampled, warped energy grid as described in Section 5. Typically, this interpolation maps the data onto a *sparser grid* and the densely sampled regions are binned before interpolation to improve the SNR, as described in Section 5. In this case, however, the non-uniformly sampled data were interpolated onto a *denser grid* instead, to prevent undersampling in certain energy regions. Based on empirical observations, we chose the heuristic of a three-times denser energy grid for interpolation.

**Figure 12:**
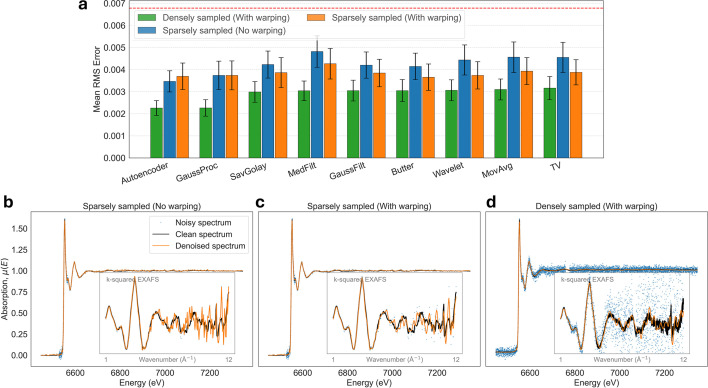
(*a*) Impact of sampling density and grid uniformity on denoising performance. Densely sampled data (green) consistently yield superior performance compared with sparsely sampled data (blue/orange), despite the lower SNR per point. For sparse datasets, the standard non-uniform acquisition protocol naturally mimics the effect of our non-stationarity warping approach, explaining the similarity in performance between warped (orange) and non-warped (blue) results. (*b*, *c*) Qualitative comparison of denoising the Mn *K*-edge of MnC_14_H_22_N_2_O_8_ using a GP denoiser. While the sparsely sampled signal exhibits a higher initial SNR compared with the densely sampled data in (*d*), the higher sampling density provides stronger constraints on local signal smoothness, resulting in better denoising performance.

First, we examine the influence of sampling density on denoising efficiency. Based on Fig. 12 ▸(*a*), densely sampled data (green bars) consistently yield superior denoising performance compared with sparsely sampled data (blue and orange bars), achieving significantly lower final RMS errors. Even though the total photon count was conserved between both datasets (resulting in a lower SNR per point for the densely sampled data), the higher sampling density provides better statistical constraints on local signal properties, such as smoothness.

This increased information density is highly beneficial for adaptive methods, such as the GP or autoencoder denoisers, because it enables them to more effectively leverage local correlations and distinguish signal from noise. In contrast, on sparsely sampled data, the performance of adaptive methods becomes similar to traditional denoisers, indicating that without sufficient local constraints even advanced methods can perform poorly. This performance difference is qualitatively illustrated in Figs. 12 ▸(*b*) and 12(*c*), where the denoised sparse spectrum exhibits larger deviations from the clean spectrum compared with densely sampled data in Fig. 12 ▸(*d*).

Moreover, regarding the sampling grid uniformity, we can observe in Fig. 12 ▸(*a*) that the denoising performance of sparsely sampled data is similar between our computational warping method (orange bars) and the non-warped data (blue bars). This similarity arises because the non-uniform data acquisition mimics the effect of our non-stationarity warping approach. These results shows that the choice of data acquisition strategy plays an important role in preconditioning the data for subsequent data processing steps, such as denoising.

In summary, while our denoising pipeline is effective for traditional step-scan XAS data, the results indicate that high-density sampling is the optimal strategy for digital post-processing. Denser sampling can minimize the risk of spectral feature undersampling and also allow methods like GPs to leverage local correlations across the energy range, resulting in more robust denoising.

### 8.5. Limitations of GP regression

Despite high denoising performance, the computational cost of GPs is high, and it scales as 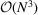 with the number of data points *N*, because of the cost of inverting the *N* × *N* covariance matrix during GP posterior prediction. The computational speed can be greatly accelerated by using graphical processing units (GPUs) rather than central processing units (CPUs) to perform the computations. While GPUs can significantly improve the computational speed, denoising of XAS spectra of size *N* requires storing multiple arrays of size *N* × *N* in GPU memory. Hence, data larger than 10000 points can quickly exceed the GPU memory of typical GPU cards with 10–20 GB of memory. Therefore, the computational complexity of GP denoising limits its use for densely sampled or time-resolved XAS data. Fortunately, in practice, many XAS spectra contain fewer than 1000 points, which allows efficient GP regression using CPUs on standard personal computers.

## 9. Impact of noise on XAS data analysis

In this section, we will discuss the impact of noise on XAS data analysis, which can be broadly categorized into analysis of XANES and EXAFS spectrum regions. We will discuss how denoising methods can improve XAS spectrum interpretation, and, finally, how to optimally measure XAS spectra.

### 9.1. EXAFS data analysis

EXAFS analysis aims at determining the local atomic structure of a material, which can be deduced from the measured absorption spectrum μ(*E*). The EXAFS signal χ(*k*) corresponds to the oscillatory component of the absorption spectrum in the post-edge region and arises from the interference between the outgoing photoelectron wave and waves scattered by neighboring atoms. To extract χ(*k*), a smooth atomic-like background μ_0_(*E*) must be estimated and subtracted from the measured absorption spectrum μ(*E*). This background subtraction step is sensitive to noise, as it requires fitting a smooth function to noisy data. In addition, subsequent EXAFS processing steps require interpolation of the data onto a uniformly sampled wavenumber *k*-grid.

**Figure 13:**
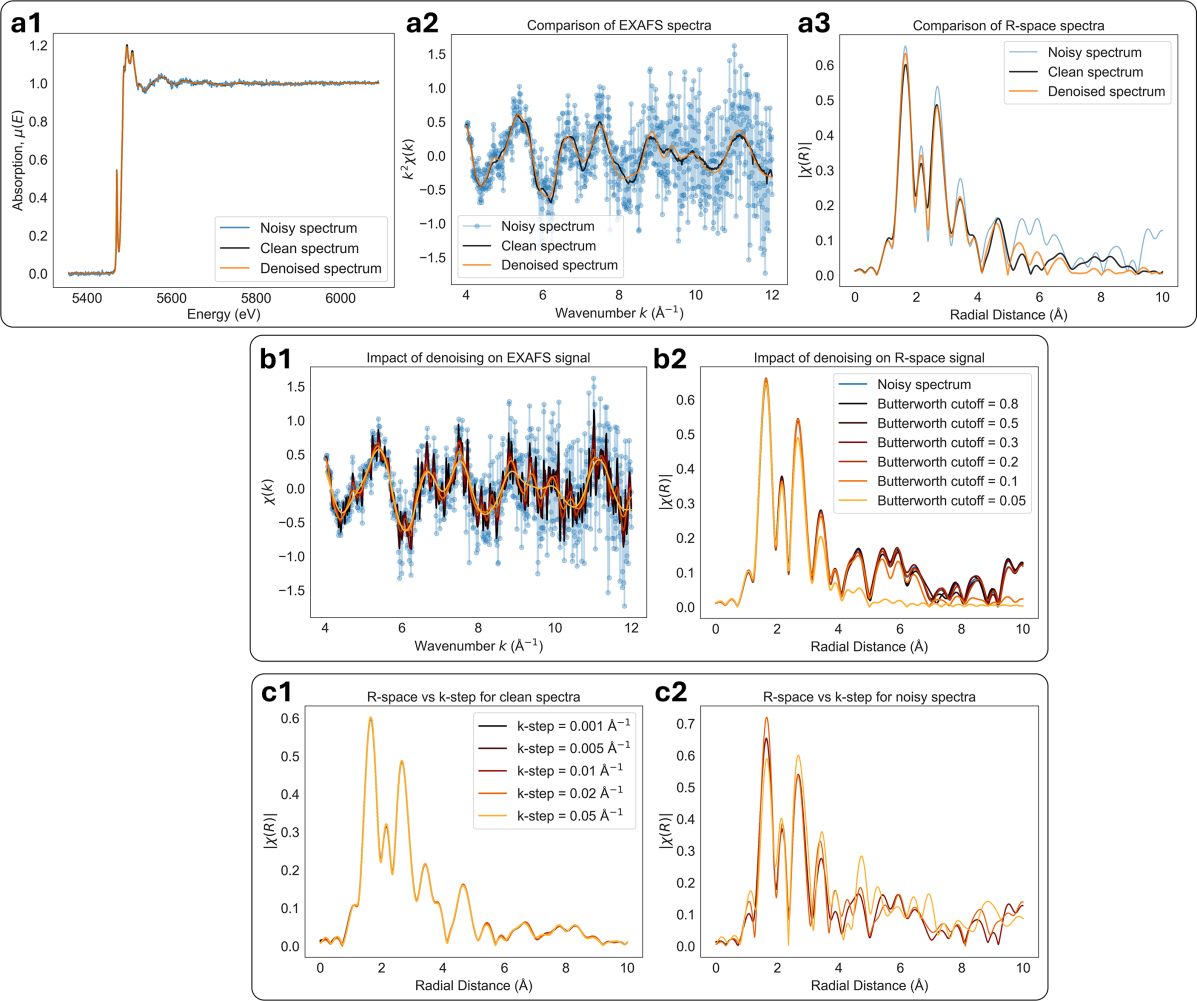
(*a*1) The measured V *K*-edge V_2_O_5_ XAS absorption spectrum μ(*E*) was denoised using a Gaussian process denoiser combined with stationarity warping. (*a*2) Comparison of *k*^2^χ(*k*) for noisy, clean, and denoised spectra. Denoising suppresses high-frequency noise while preserving the underlying EXAFS oscillations. (*a*3) Corresponding Fourier-transformed EXAFS magnitude |χ(*R*)|, showing that denoising reduces noise-related artifacts at larger *R* without introducing additional peaks in the first coordination shells. (*b*1, *b*2) Morever, denoising tends to not introduce additional features within the χ(*k*) and χ(*R*) signals. By varying the Butterworth denoiser cutoff frequency, we demonstrate that denoising suppresses existing features without introducing additional peaks. (*c*1, *c*2) Effect of different uniform *k*-grid step sizes on |χ(*R*)| for a clean spectrum and noisy spectrum. Coarser *k*-grid sampling leads to significant distortions due to interpolation artifacts for noisy data, highlighting the importance of appropriate *k*-grid selection when analyzing noisy EXAFS data.

Once χ(*k*) is obtained, it can be Fourier transformed to obtain χ(*R*), where *R* represents the radial distance from the absorbing atom. Peaks in χ(*R*) correspond to contributions from atomic scatterers at different effective distances and provide information about the local coordination environment. We demonstrate the effect of noise for χ(*R*) analysis in Fig. 13 ▸ by comparing results of clean, noisy, and denoised experimental XAS spectra shown in Fig. 13 ▸(*a*). Based on Fig. 13 ▸(*a*2), denoising is able to significantly reduce noise of the χ(*k*) signal. Looking at χ(*R*) shown in Fig. 13 ▸(*a*3), we observe that the impact of noise is relatively minor for the first few coordination shells below *R* < 5 Å and is mostly impacting the higher coordination shells by introducing non-physical oscillations. By using denoising, the quality of χ(*R*) improves for shells below *R* < 5 Å, and the noise-induced oscillations beyond *R* > 5 Å are significantly suppressed.

In practice, χ(*R*) is relatively robust to uncorrelated noise. That is because χ(*k*) consists of a broad, low-frequency oscillatory signal, while noise typically occupies the higher frequencies. After the Fourier transform, such noise appears primarily as a smooth background or increased noise floor in *R*-space. As a result, noise mainly affects higher coordination shells at large distances (typically *R* > 5 Å), while the first few coordination shells remain largely unaffected. Moreover, as shown in Figs. 13 ▸(*b*1)–13(*b*2), denoising tends not to introduce additional peaks into the χ(*R*) signal and instead suppresses features that are already present. By varying the Butterworth denoiser cutoff frequency parameter, the corresponding χ(*R*) spectral features get suppressed without the introduction of artifacts.

Moreover, when dealing with noisy χ(*k*), the choice of the uniform *k*-grid used for interpolation becomes especially important. In standard XAS processing workflows, many commonly used software packages, such as *Larch* (Newville, 2013 ▸) or *Athena* (Ravel & Newville, 2005 ▸), use a fixed default *k*-grid sampling of around *k*_step_ = 0.05 Å^−1^. In practice, however, quick-EXAFS beamlines (*e.g.* SuperXAS or Debye at the Swiss Light Source) can acquire data on significantly denser energy grids, which require much finer *k*-space sampling.

While interpolation onto a coarser *k*-grid has little impact for high SNR data, it can strongly affect noisy χ(*k*) signals. Interpolating noisy data onto an undersampled *k*-grid can distort the oscillatory structure of χ(*k*), suppress high-frequency components, and introduce correlations that are not present in the original measurements. These effects propagate into the Fourier-transformed χ(*R*), where they appear as artifacts distorting all of the coordination shells. The impact of incorrect *k*-grid sampling is illustrated in Figs. 13 ▸(*c*1)–13(*c*2), where we compare interpolation onto undersampled and properly sampled *k*-grids, while all other processing parameters were kept fixed. For noisy spectra, coarser *k*-grid interpolation leads to visibly degraded χ(*k*) and increased artifacts in χ(*R*) as shown in Fig. 13 ▸(*c*2), whereas finer *k*-grid sampling preserves the oscillatory structure and yields more stable *R*-space representations. This highlights the importance of carefully selecting the interpolation grid when processing noisy EXAFS data, particularly in high-speed or low-SNR measurement regimes.

The optimal *k*-grid sampling can be calculated from the measured energy grid by differentiating the expression for the wavenumber *k* in equation (5) with respect to the energy 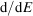. In doing so, we obtain the following relationship, 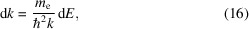
which states that the *k*-grid spacing d*k* is proportional to the energy grid spacing d*E* and inversely propotional to *k*. Therefore, the optimal (smallest) uniform sampling step Δ*k* is determined by the maximum wavenumber *k*_max_ and the minimum energy step Δ*E*_min_,
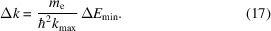
In addition to χ(*R*) analysis, an alternative and widely used approach is fitting the EXAFS equation to χ(*k*) in order to extract structural parameters such as interatomic distances, coordination numbers, and disorder parameters. While noise can directly affect the stability and uncertainty of such fits, EXAFS fitting is an inherently ill-posed inverse problem, in which multiple parameter combinations can produce similarly good fits to the data. As a result, fitting quality depends strongly on user-defined parameter choices, making it difficult to attribute differences in fitting quality between noisy and denoised spectra to noise reduction alone. Therefore, we refrained from EXAFS fitting.

### 9.2. XANES data analysis

XANES analysis focuses on the near-edge region of the absorption spectrum and provides information about the electronic structure, oxidation state, and local coordination geometry of the absorbing atom. In contrast to EXAFS, XANES features are typically sharper and the overall SNR is higher due to the stronger absorption cross-section. However, the characteristic spectral features in XANES often have widths (or frequencies) that are comparable with those of the experimental noise. Therefore, low-SNR measurements make XANES interpretation difficult, as is often the case for operando experiments.

XANES analysis is commonly used for operando and time-resolved studies, where the goal is to track and interpret the chemical evolution of a reaction based on spectral changes. To identify these changes, fitting and decomposition methods are used, such as linear combination analysis (Newville, 2014 ▸; Ravel & Newville, 2005 ▸; Calvin, 2013 ▸), principal component analysis (Wasserman, 1997 ▸), and multivariate curve resolution (MCR) (Tauler, 1995 ▸; Wasserman, 1997 ▸; Camp Jr, 2019 ▸). When dealing with low-concentration samples or high-speed measurements, noise can obscure subtle spectral differences and degrade the stability of the spectral decomposition. For example, MCR is highly sensitive to both the SNR of the input data and the choice of initial estimates for the spectral components. Poor initial guesses or high noise levels can lead to slow convergence, unstable solutions, or non-physical decomposition results. As a result, MCR analyses of noisy operando XANES data often require additional preprocessing steps, such as temporal binning, which reduces time resolution.

**Figure 14:**
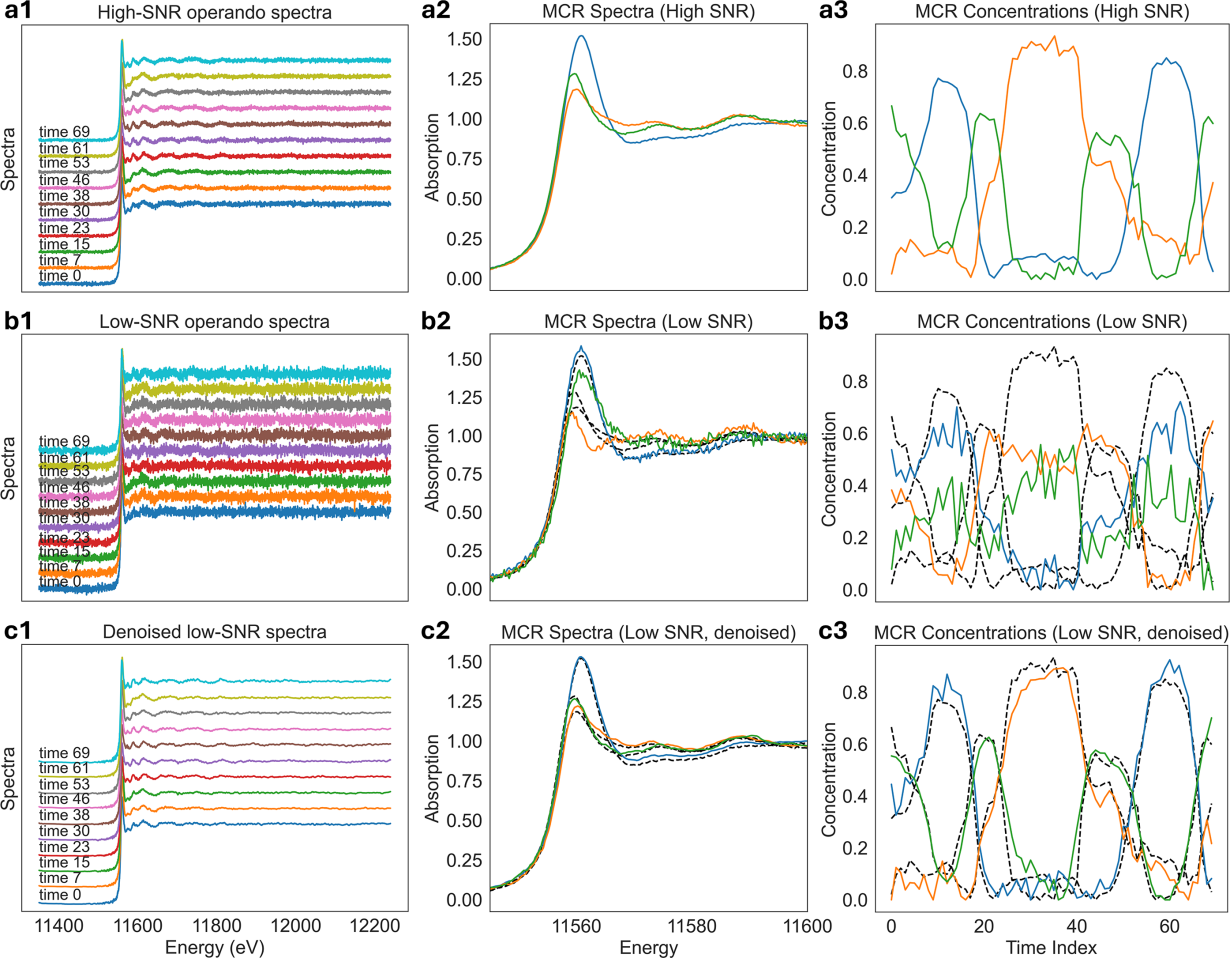
(*a*1) High-SNR operando XANES dataset and corresponding MCR results. (*a*2) The corresponding MCR-extracted component spectra and (*a*3) corresponding concentration profiles as a function of time. (*b*1) Low-SNR operando dataset obtained by using ten times fewer spectra for temporal binning compared with the high-SNR data. (*b*2) The corresponding MCR component spectra and (*b*3) resulting concentration profiles, both of which exhibit non-physical spectra and temporal dynamics. Dashed black curves indicate the high-SNR reference concentrations for comparison. (*c*1) Same low-SNR dataset after denoising, which results in physically correct (*c*2) MCR component spectra and (*c*3) concentration profiles, showing substantially improved agreement compared with high-SNR spectra. These results demonstrate that denoising improves the robustness and stability of MCR analysis for noisy operando XANES measurements without introducing additional spectral features. The Pt *L*_3_-edge XAS operando spectra were denoised using a 2D moving average filter along both temporal and energy axes, together with stationarity warping.

We demonstrate in Fig. 14 ▸ that denoising prior to spectrum decomposition can significantly improve MCR performance. From experimental Pt *L*_3_-edge XAS operando spectra, we obtained a high-SNR dataset by temporally binning ten neighboring spectra shown in Fig. 14 ▸(*a*1). MCR was used to decompose the spectra into three spectral components, which are shown in Fig. 14 ▸(*a*2), together with the corresponding concentration profiles in Fig. 14 ▸(*a*3). We used the *PyMCR* (Camp Jr, 2019 ▸) package together with the *SIMPLISMA* (Windig & Stephenson, 1992 ▸) algorithm to generate initial spectral estimates. To demonstrate how noise affects MCR analysis, we created a low-SNR dataset, shown in Fig. 14 ▸(*b*1), by selecting every tenth temporal instance, which results in a low-SNR dataset with a ten-times lower integration time compared with the high-SNR dataset. As shown in Figs. 14 ▸(*b*2)–14(*b*3), due to the lower SNR, the MCR decomposition can no longer obtain physically meaningful spectral components and their concentration profiles.

By denoising the low-SNR data, we obtain a clear improvement in signal quality, as shown in Fig. 14 ▸(*c*1). This leads to a more reliable estimation of the initial spectral components and, in turn, to more stable and physically meaningful MCR decomposition results [Fig. 14 ▸(*c*2)–14(*c*3)]. Therefore, denoising improves both the component initialization and the robustness of the final MCR solution, enabling physically meaningful component separation without the need for temporal binning. In this example, denoising yields decomposition results comparable with those obtained from the high-SNR dataset, which required a tenfold increase in effective integration time and thus a tenfold loss in temporal resolution. While MCR itself performs a form of noise suppression through data decomposition, additional denoising becomes increasingly important when the SNR is low or when the ratio between the number of time instances and the number of spectral components decreases. Therefore, denoising becomes critical when pushing the limits of high-speed operando XAS measurements.

### 9.3. Sampling considerations for XAS acquisition

For standard XAS analysis approaches, the fundamental information content is determined by the accessible *k*-range and the SNR rather than by the exact sampling density, provided that the spectrum is sufficiently sampled. In the presence of Poisson noise, the SNR scales with the square root of the total number of detected photons. As a result, distributing photons across a denser *k*-grid with shorter integration times [Fig. 9 ▸(*a*)], or concentrating them into fewer points with longer integration times [Fig. 9 ▸(*b*)], will yield comparable information content from the point of view of XAS analysis.

In practice, however, the choice of sampling density can significantly influence denoising performance. As discussed in Section 8.4 and demonstrated by Fig. 12 ▸, methods that exploit local correlation structure, such as GP or deep-learning-based denoisers, strongly benefit from denser sampling grids. These methods leverage the increased information density to better constrain the inferred signal and distinguish it from noise. For other denoising methods, such as convolution-based filters, performance is primarily governed by the overall photon statistics and the SNR. Since densely measured signals can always be computationally binned onto a sparser grid post-acquisition, high-density sampling is superior in terms of denoising performance and greater post-processing flexibility.

**Figure 15:**
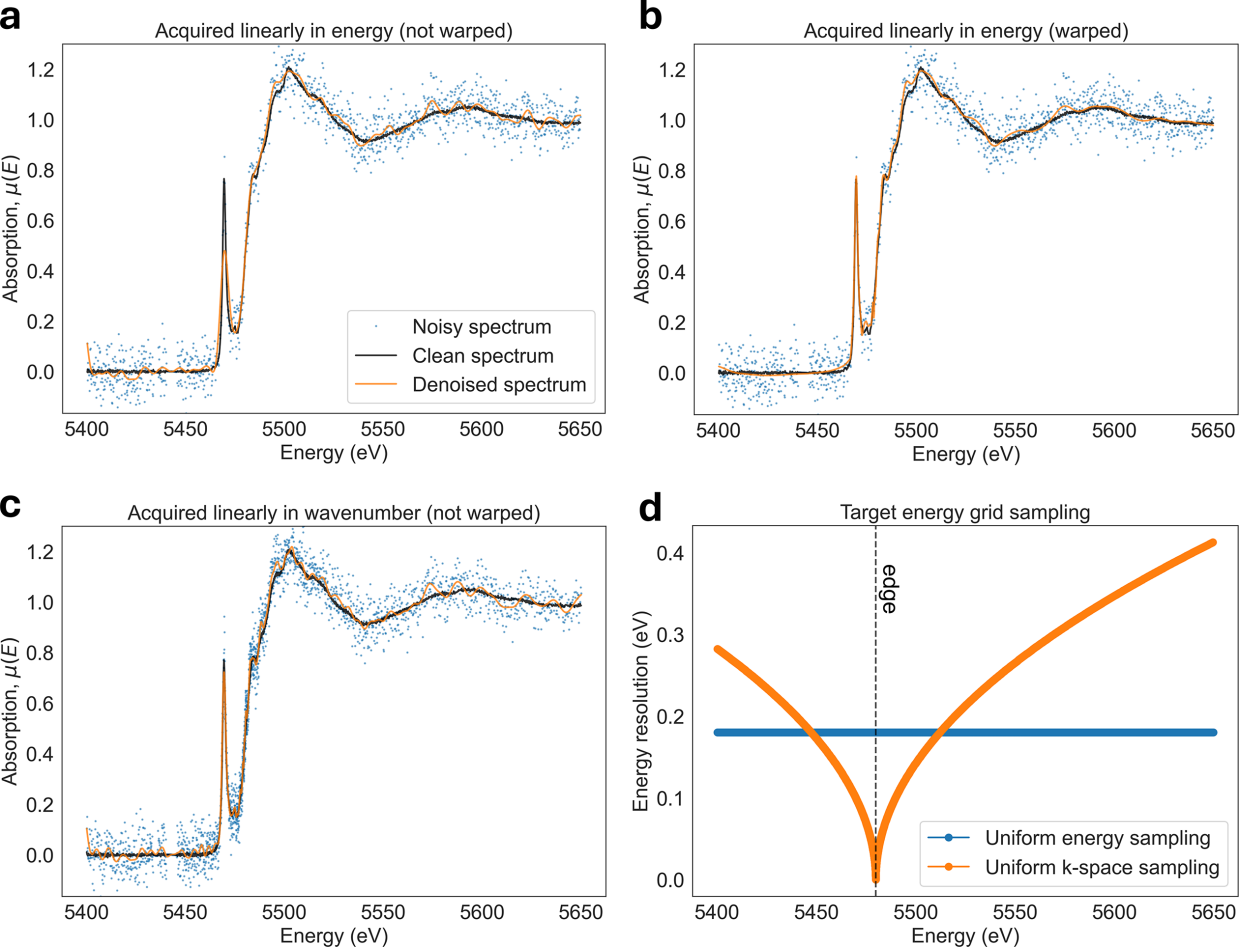
(*a*, *b*) By denoising warped rather than non-warped data, denoising performance is greatly improved. (*c*) An alternative approach is to collect XAS data on a grid where it appears stationary, and warping is no longer necessary. One example of data sampling is shown in (*d*) where the data acquisition follows uniform *k*-grid sampling before and after the absorption edge. While such sampling does not have physical meaning for the pre-edge region, it provides a way to densely sample sharp pre-edge peaks and then gradually reduce sampling density for the featureless pre-edge region. The V *K*-edge BiVO_4_ XAS spectrum was denoised using a Butterworth denoiser and an XAS warping function.

In addition to sampling density, the uniformity of the sampling grid itself is important. Based on the analysis in Section 8.4, the optimal data acquisition is onto an energy grid that follows uniform sampling in *k*-space (wavenumber). As described in Section 4.3, such a grid produces XAS signals that appear effectively stationary, resulting in significantly improved denoising performance without the need for additional computational processing (Fig. 15 ▸). Measurements onto uniformly sampled *k*-grids are already widely used for XAS experiments to facilitate subsequent EXAFS analysis steps, which require interpolation onto a uniform *k*-space grid. Our work provides an alternative motivation for this strategy, demonstrating that it can provide significantly improved denoising performance.

Therefore, we conclude that high-density sampling on grids uniform in *k*-space is the best strategy, facilitating superior signal denoising. While sparse sampling with long integration times is sufficient for basic XAS analysis, it loses the ability to capture local signal correlations and can undersample fine spectral features. As a result, it is best to increase sampling density at the expense of reduced SNR per point, as this maximizes the effectiveness of modern denoising algorithms.

### 9.4. Impact of noise on data pre-processing

Many XAS preprocessing steps, such as pre- and post-edge normalization or estimation of the smooth atomic background, are sensitive to measurement noise. This is because these steps typically rely on fitting routines, which can be strongly affected by noise and outliers. In addition to random noise, experimental XAS data can contain monochromator glitches, which appear as sharp and relatively broad intensity artifacts. These glitches are difficult to remove manually, especially when they overlap with spectral features. Simple removal of glitch regions can introduce discontinuities in the spectrum, which can negatively affect subsequent processing steps and analysis, including EXAFS background subtraction and fitting.

**Figure 16:**
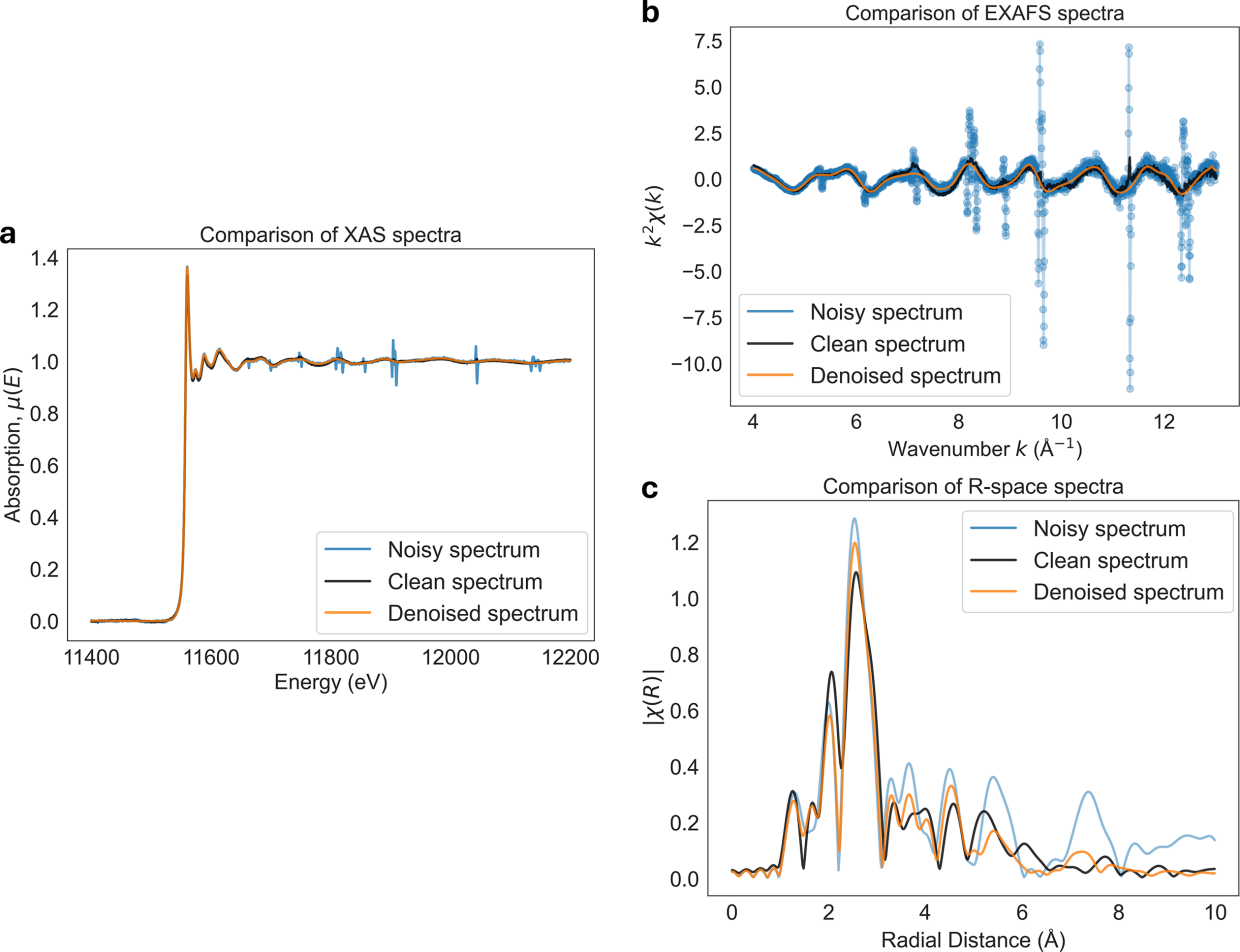
(*a*) Comparison of Pt *L*_3_-edge absorption spectra μ(*E*) measured in fluorescence mode (clean reference) and transmission mode (noisy, with glitches). The transmission XAS spectrum was denoised to illustrate glitch mitigation. (*b*) Corresponding *k*^2^χ(*k*) spectra, showing high-frequency glitches which are greatly suppressed by denoising. (*c*) The magnitude of the Fourier-transformed EXAFS signal χ(*R*) demonstrates that denoising can recover peaks associated with the main coordination shells while also suppressing noise at higher radial distances. The spectrum was denoised using a Gaussian process denoiser combined with stationarity warping.

Denoising provides a practical and automated approach to reduce both random noise and glitch-related artifacts prior to further data processing. Fig. 16 ▸ illustrates an example using experimentally measured Pt *L*_3_-edge XAS data. In Fig. 16 ▸(*a*), the absorption spectrum μ(*E*) acquired in transmission mode contains strong glitches, while a corresponding clean reference spectrum was obtained from a fluorescence measurement of the same sample. After denoising, the transmission spectrum closely matches the clean reference, while preserving the underlying spectral features. The noisy spectrum demonstrates the effects of glitches, which propagate into the *k*^2^χ(*k*) EXAFS signal shown in in Fig. 16 ▸(*b*) and the Fourier-transformed EXAFS signal χ(*R*) shown in Fig. 16 ▸(*c*).

Denoising can suppress glitches and also recover the smooth oscillatory EXAFS features [Fig. 16 ▸(*b*)] without the need for manual glitch region selection and their deletion, which can lead to discontinuities. Moreover, denoising can also improve the quality of the χ(*R*) signal [Fig. 16 ▸(*c*)] by reproducing the main coordination shells below *R* < 4 Å of the clean reference without introducing additional features. In addition, denoising also removes spurious oscillations and artificial peaks at larger radial distances *R* > 4 Å without introducing additional features

In practice, denoised spectra can be used to obtain more robust estimates of normalization and background functions, which can then be applied to the original noisy data. Alternatively, the denoised spectra themselves can be used directly for XAS analysis. This example demonstrates that denoising can effectively suppress artifacts due to noise and glitches in both χ(*k*) and χ(*R*), improving the reliability of XAS preprocessing and analysis.

## 10. Conclusion

In this work, we introduce a flexible and automated framework for denoising XAS data, addressing key challenges such as signal non-stationarity and irregular sampling. A key innovation of our denoising pipeline is the concept of stationarity warping, a preprocessing step that transforms XAS data from the energy domain into a uniform space where spectral features become stationary. This significantly improves the performance of both traditional and modern denoising methods. We show that even simple methods like Butterworth or moving average filters significantly benefit from stationarity warping, allowing them to perform similarly to modern and more complicated denoising approaches. Our denoising pipeline also includes specialized methods for time-resolved spectrum denoising that leverage both spatial and temporal correlations across neighboring time instances. These include 2D window-based approaches, where asymmetric windows are applied with different extents along the spatial and temporal axes.

The intuition behind the warping approach is that typical denoising filters effectively act as low-pass filters with a fixed bandpass applied uniformly across the signal. This is undesirable when the characteristic feature width of the signal varies across the input domain. Addressing this would normally require adaptive denoising methods capable of locally adjusting their low-pass filtering properties. Our stationarity warping method provides a simple alternative, which, by transforming the data into a domain where features appear stationary, allows any denoising method to behave as a variable low-pass filter without modifying the underlying denoising algorithm.

We also demonstrate that Gaussian process and convolutional autoencoder-based denoisers outperform traditional filters, especially for highly noisy XAS signals. With our stationarity warping methodology, we demonstrated that the GP denoiser can be successfully used to denoise a broad variety of XAS signals. Together with the automated and simplified GP optimization strategy, we provide a fully automated GP-based data denoising framework. Our convolutional autoencoder denoiser trained using the *Noise2Noise* framework was able to generalize well across unseen spectral compounds and different signal sampling conditions compared with the training data. This generalization is offered by our stationarity warping methodology, which allows standardization of XAS data from a broad variety of experimental conditions to match the training data more closely. Moreover, we demonstrate that *Noise2Noise* is an effective method for convolutional autoencoder denoiser training, when noise-free XAS reference spectra are not available. Instead, with the *Noise2Noise* framework we can train high-performance models using multiple repeated noisy XAS spectrum measurements.

We also examined the impact of denoising on various aspects of XAS data analysis, including pre-processing, EXAFS analysis, and XANES-based spectral decomposition. Our results show that while some standard analysis steps, such as Fourier transformation of χ(*k*), are relatively robust to moderate levels of noise, denoising becomes increasingly important for low-SNR operando measurements.

In general, denoising improves the reliability of preprocessing steps such as normalization and background subtraction, reduces glitch-induced artifacts that can propagate into χ(*k*) and χ(*R*), and stabilizes multivariate decomposition methods used for operando XANES analysis. Importantly, our examples demonstrated that denoising methods tend not to introduce additional spectral features such as non-physical coordination shells. Together, these results indicate that denoising can improve robustness and interpretability of XAS analysis.

In summary, we present a comprehensive XAS denoising pipeline that integrates a wide range of denoising techniques and introduces a novel signal warping approach capable of enhancing the performance of any denoising method. The pipeline includes newly developed Gaussian process-based denoiser and a supervised convolutional autoencoder denoiser, which was trained on experimental rather than simulated data. All methods are implemented in our open-source Python package, available on GitHub (https://github.com/tomasaidukas/XASDenoise). This work lays a foundation for future developments, such as self-supervised real-time denoising during data acquisition, unsupervised learning of warping functions, and extension to other spectroscopic modalities beyond XAS.

## Supplementary Material

The link contains: - Experimental XAS spectra dataset - Denoising software used for data benchmarking, together with the associated Python scripts used to generate the figures and results within the manuscript.: https://doi.org/10.5281/zenodo.17434349

## Data Availability

The *XASDenoise* Python-based denoising software is publicly available on GitHub (https://github.com/tomasaidukas/XASDenoiseXASDenoise).We also provide the training and testing experimental XAS datasets, the denoising code, and benchmarking scripts on Zenodo (https://doi.org/10.5281/zenodo.17434349DOI).

## Appendix A Implementation details

Our denoising pipeline is based on the Python programming language. All steps within the denoising pipeline are modular, allowing for easy modification and replacement of various parts within the pipeline. Hence, while our software is focused on XAS data denoising, it can also be used for the denoising of other signals from other spectroscopies. The main requirement is that the signal passed to the pipeline is either a single XAS spectrum (1D array) or a time-series measurement (2D array) from, for example, *in situ* or *operando* XAS experiments. Our denoising pipeline can be generalized as having two functions: signal pre-processing and signal denoising. The pre-processing steps include methods such as edge-step removal, noise estimation as well as the stationarity warping.

Once processed, the data are passed to a denoising class. Each denoiser class is responsible for interpreting the inputs provided by the pipeline (*e.g.* warped or uniformly resampled data), applying the denoising algorithm with user-defined denoising parameters, and returning the denoised output in a format compatible with downstream steps such as unwarping and post-processing. This structure ensures that all denoisers (traditional filtering methods or trained neural network models) can be used interchangeably within the same pipeline without modification of the workflow.

The current implementation contains three separate denoising classes for *Gaussian process denoising*, *convolutional autoencoder denoising*, and *traditional denoising*. While the contents of each denoiser class can vary, each class must contain a method named *denoise*, which is called by the denoising pipeline to perform signal denoising. Such modular design allows for seamless integration of a wide range of denoising algorithms, each encapsulating its own logic for data handling and processing.

The traditional denoiser class contains multiple denoising methods, which were implemented from existing Python libraries such as *scikit-image* (van der Walt *et al.*, 2014 ▸), *Scipy* (Virtanen *et al.*, 2020 ▸) and *PyWavelets* (Lee *et al.*, 2019 ▸). The optimization required by the Gaussian process denoiser is performed by the *GPyTorch* library (Gardner *et al.*, 2021 ▸), which is based on a popular machine learning library called *PyTorch* (Paszke *et al.*, 2019 ▸), offering GPU-accelerated optimization methods. The autoencoder-based denoiser training and inference steps are implemented using *PyTorch*.

## Appendix B Traditional denoisers

Here, we introduce the traditional denoisers implemented within our pipeline, which aim to recover a denoise signal  from noisy measurements *y*.

**Moving average filter**: smooths the signal using a symmetric window of size 2*w* + 1,

**Gaussian filter**: applies a weighted average with a Gaussian kernel defined by standard deviation σ at every signal point *y*_*i*_,

**Savitzky-Golay filter**: fits a polynomial of degree *d* over a moving window of size 2*w* + 1 to preserve higher-order features,

**Median filter**: replaces each signal point *y*_*i*_ with the median within a moving window of size 2*w* + 1,

**Butterworth filter (low-pass)**: suppresses high-frequency components with a smooth frequency response,

where *f*_c_ is the cutoff frequency, *n* is the filter order,  is the Fourier transform operator and *H*(*f*) is the low-pass filter transfer function.

**Wavelet denoiser**: decomposes the signal **y** using wavelets ψ_*k*_ and thresholds the coefficients *c*_*k*_,

The denoised signal is reconstructed from the thresholded coefficients ,

Key parameters include the wavelet type (*e.g.* Daubechies, Symlets), decomposition level *k* and threshold operator *T*. Thresholding is achieved by manually selecting only a specific number of coefficients to keep for signal recovery.

**Total variation denoiser**: minimizes signal variations while preserving key features by solving

where λ controls the trade-off between fidelity and smoothness.

Each method has one or more parameters (*e.g.**w*, σ, λ, *T*, *d*) that must be tuned. For example, window size *w* or Gaussian filter σ control the level of signal smoothing. Automated selection of optimal denoiser parameters is described in Appendix *B*1 [App appb].

### b1. Denoiser parameter optimization

Selecting optimal denoising parameters can be a difficult and time-consuming task, particularly for users who are unfamiliar with the internal logic of a given denoising method. To automate parameter selection, we minimize a total variation (TV) loss function defined as

where *y* is the noisy signal,  is the denoised signal, **p** =  are the parameters of the denoiser, λ is the weight of the TV penalty and σ is the signal noise level estimate. The data fidelity term avoids oversmoothing by ensuring that the denoised signal is similar to the noisy input. However, this term alone is insufficient because it will be minimized by performing no denoising at all. In contrast, the TV term ensures the smoothness of the denoised signal by penalizing sharp gradients. Therefore, minimization of equation (18) recovers denoising parameters **p**, which achieves a compromise between noise suppression and feature preservation. The only parameter that needs to be tuned is λ, which we scale by the estimated signal noise level σ. This allows optimal denoiser parameter selection for signals across varying noise levels automatically. For all denoising methods displayed in this manuscript, λ was set to 0.002. We minimize the loss in equation (18) either by performing a grid search across a number of candidate parameters or by performing gradient descent optimization (Ruder, 2016 ▸). To avoid convergence issues, all denoiser parameters in this manuscript were optimized using the grid search method across 50 candidate parameters.

## Appendix C GP regression details

### c1. GP model

A GP model defines a distribution over functions *f*(*x*), meaning it represents all the plausible smooth curves that could explain the observed data. Instead of choosing a single function to fit the data, the GP framework considers a probability distribution of all possible functions that are consistent with the signal characteristics (*e.g.* smoothness, correlation length).

### c2. Covariance function (kernel)

The kernel *k*(*x*, *x*′) encodes assumptions about the smoothness and structure of the signal. We use the Matérn class of kernels, which is well suited for modeling XAS spectra due to its ability to capture both sharp features and varying levels of smoothness. The general form of the Matérn kernel for  is

where ℓ is the characteristic lengthscale corresponding to the feature width within the XAS signal,  is the kernel amplitude, Γ(·) is the gamma function, *K*_ν_(·) is the modified Bessel function of the second kind and ν controls the smoothness (larger ν corresponds to smoother functions). The commonly used values for ν are:

(i)  = 1/2 gives the exponential kernel;

(ii)  = 3/2 gives once-differentiable functions;

(iii)  = 5/2 gives twice-differentiable functions.

The lengthscale ℓ and scaling σ_*f*_ hyperparameters can be learned from the data during GP regression.

### c3. Posterior prediction

Once the kernel function *k*_Matern_(*x*, *x*′) and the noise prior  are defined, the GP uses the observed noisy data to infer the most likely underlying signal. Specifically, it computes the posterior distribution over the function *f*(*x*) given the data. The mean of this posterior, μ_*_(*x*), serves as the denoised signal,

where

(i) **K**_*NN*_ is the kernel matrix between training inputs *x*;

(ii) **K**_**N*_ is the kernel between test points *x*_*_ and training points;

(iii)  =  is the noise variance;

(iv) **y** is the observed noisy spectrum.

This equation computes a weighted average of the observed data, where the weights are determined by both the kernel and the noise prior. In practice, this means that regions of the spectrum with low noise will be trusted more, and regions with high noise will be smoothed more aggressively. The result is a signal that retains important spectral features while suppressing noise, with uncertainty estimates provided by the GP variance.

### c4. Hyperparameter optimization

The kernel parameters, such as the lengthscale ℓ and amplitude σ_*f*_, are automatically learned from the data during optimization. This is achieved by maximizing the marginal likelihood of the observed noisy signal for a given GP model. In practice, this step balances how well the model fits the data against how complex the model are. To perform the GP regression, we use a Python-based library called *GPyTorch* (Gardner *et al.*, 2021 ▸), which provides a broad range of kernel functions and optimization strategies. We have packaged the necessary *GPyTorch* optimization functionality into an automated XAS data denoising framework.

### c5. Heteroskedastic noise estimation

For XAS signals, the common assumption is that the noise is heteroskedastic, that is, the noise variance varies across the energy domain and depends on the signal input . Since GPs use the noise variance to differentiate signal from the noise, noise values that are too high will lead to signal oversmoothing (*i.e.* signal will be estimated as noise). Therefore, we must select the lower confidence bound on the noise estimate to perform optimal signal denoising.

It is natural to assume that the noise variance cannot change too dramatically over the inputs. Therefore, we try to learn a smooth function  that approximates the lower bound of the real noise variance with high probability. We begin by assuming that within short windows  =  the noise level is constant, allowing the use of simple stationary GP regression to estimate a noise-free signal μ_GP_(*x*). By performing GP regression for each of these narrow windows denoted by index *i*, we can compute the residual between the noisy data and the noise-free estimate Δ_*t*_ = *y*_*t*_ − μ_GP_(*x*_*t*_), where . Assuming that each window  contains *N* samples, we can estimate the sample variance of the noise  as the following random variable  = . In doing so, we assume that  must be distributed according to the χ^2^ distribution (Casella & Berger, 2002 ▸) with *N* degrees of freedom. This assumption allows us to construct a confidence interval for the real value of the noise , which is valid only within a given interval . To avoid signal oversmoothing we take the lower bound, which implies that, for *N* = 10, our lower bound noise standard variance estimator is . If these estimators are still highly non-smooth, we smooth them using a moving average filter.
